# Inhibition of endocytic lipid antigen presentation by common lipophilic environmental pollutants

**DOI:** 10.1038/s41598-017-02229-7

**Published:** 2017-05-18

**Authors:** Manju Sharma, Xiang Zhang, Shuangmin Zhang, Liang Niu, Shuk-mei Ho, Aimin Chen, Shouxiong Huang

**Affiliations:** 1 Division of Environmental Genetics and Molecular Toxicology, Department of Environmental Health, University of Cincinnati College of Medicine, Cincinnati, OH 45267 USA; 2 Genomics, Epigenomics and Sequencing Core, Department of Environmental Health, University of Cincinnati College of Medicine, Cincinnati, OH 45267 USA; 3 Division of Biostatistics and Bioinformatics, Department of Environmental Health, University of Cincinnati College of Medicine, Cincinnati, OH 45267 USA; 4 Division of Epidemiology, Department of Environmental Health, University of Cincinnati College of Medicine, Cincinnati, OH 45267 USA

## Abstract

Environmental pollutants as non-heritable factors are now recognized as triggers for multiple human inflammatory diseases involving T cells. We postulated that lipid antigen presentation mediated by cluster of differentiation 1 (CD1) proteins for T cell activation is susceptible to lipophilic environmental pollutants. To test this notion, we determined whether the common lipophilic pollutants benzo[a]pyrene and diesel exhaust particles impact on the activation of lipid-specific T cells. Our results demonstrated that the expression of CD1a and CD1d proteins, and the activation of CD1a- and CD1d-restricted T cells were sensitively inhibited by benzo[a]pyrene even at the low concentrations detectable in exposed human populations. Similarly, diesel exhaust particles showed a marginal inhibitory effect. Using transcriptomic profiling, we discovered that the gene expression for regulating endocytic and lipid metabolic pathways was perturbed by benzo[a]pyrene. Imaging flow cytometry also showed that CD1a and CD1d proteins were retained in early and late endosomal compartments, respectively, supporting an impaired endocytic lipid antigen presentation for T cell activation upon benzo[a]pyrene exposure. This work conceptually demonstrates that lipid antigen presentation for T cell activation is inhibited by lipophilic pollutants through profound interference with gene expression and endocytic function, likely further disrupting regulatory cytokine secretion and ultimately exacerbating inflammatory diseases.

## Introduction

Whereas genetic variants are the major determinants of Mendelian disorders, various non-heritable environmental pollutants are increasingly recognized as important factors causing severe pathology in complex diseases^1–3^. In allergic, autoimmune, and tumorigenic inflammations, human-environmental interaction is crucial in affecting the gene expression and protein function in pathogenic processes^3, 4^. Benzo[a]pyrene (BaP), a prototypic polycyclic aromatic hydrocarbon (PAH), is a common environmental pollutant that impacts human life on a daily basis. Humans are constantly exposed to PAHs released from fuel-burning vehicles, industrial gas discharge, and cigarette smoking, and even contained in the grilled, barbecued, and smoked foods^5^. Environmental pollutants, such as BaP and other PAHs, have been considered to be critical causative or risk factors for triggering or aggravating multiple inflammatory diseases and conditions, including allergic inflammations^6^, obesity^7^, intrauterine growth restriction, and preterm birth^8, 9^. Moreover, PAHs also exacerbate cardiovascular, lung, and autoimmune diseases and cancers^10^. PAHs and many other environmental pollutants are considered lipophilic because of their chemical ability to dissolve in organic solvents^11^, associate with lipid-enriched tissues^7^, and potentially interact with the hydrophobic domains of protein receptors^12^. The process by which lipophilic pollutants target innate and adaptive immune systems to disrupt the homeostasis of inflammatory responses and trigger multiple diseases remains elusive. We are interested in understanding whether lipophilic PAH pollutants and especially BaP impact the presentation of lipid antigens and activation of lipid-specific T cells.

Lipid-specific T cells, such as natural killer T (NKT) cells, are T cell populations activated by CD1-presented lipid antigens^13^. Lipid-specific T cells were shown to be abundant in the peripheral blood of healthy humans and up to 10% of total T cells were considered autoreactive lipid-specific T cells, supporting an important immune regulatory mechanism in health and disease^14–16^. Although it remains elusive for the functions of different subsets of lipid-specific T cells due to undefined self-lipid antigens and a lack of detection reagents, it is known that autoreactive lipid-specific T cells are readily responsive for cytokine secretion and effector function in health and disease^17^. Indeed, the regulatory function of lipid-specific T cells has been shown in microbial infections and inflammatory conditions^13, 18, 19^. Specifically, these cells are critical in different inflammatory conditions that are sensitive to exposure to environmental pollutants. For example, lipid-specific T cells have a protective role against obesity by secreting anti-inflammatory cytokines^20^. NKT cells are abundant and regulatory in multiple conditions, including normal pregnancy^21^, lung inflammation^22, 23^, liver inflammation^24^, and cancers^25^. Thus, NKT cells and other lipid-specific T cells are crucial T cell populations with multiple subsets and heterogeneous functions in the regulation of various inflammatory diseases^18^.

Unlike conventional T cells, which are restricted by highly polymorphic major histocompatibility complex (MHC) molecules, lipid-specific T cells are activated by non-polymorphic CD1 proteins through lipid antigen presentation. As characterized over the last two decades, lipid antigen presentation is a cellular pathway by which CD1 proteins load lipid metabolites, express onto the cell surface, and interact with T cell receptors for T cell activation^26, 27^. Human dendritic cells (DCs) express CD1a, CD1b, CD1c, and CD1d proteins, among which CD1c and CD1b are used as markers for defining conventional DCs^27, 28^. Lipid metabolites are preferentially loaded to CD1 proteins in different endocytic compartments, for example, in early or recycling endosomes for CD1a and late endocytic compartments for CD1d^26, 29^. Unlike peptide antigen presentation^30^, lipid antigen presentation requires lipid metabolism to provide lipid metabolites^15, 31–33^, lipoproteins for lipid transportation, chaperoning proteins for intracellular lipid transfer, and lipid association with CD1 proteins^13, 34^. Biochemically, these processes consist of abundant hydrophobic lipid-protein interactions, which can be potentially targeted by lipophilic pollutants similar to the pollutant interactions with aryl hydrocarbon receptors (AHR), hormone receptors, and other transcription factors^4, 10, 12^. In this speculative context, determining the actual impact of lipophilic pollutants on lipid antigen presentation will be interesting.

In this study, we demonstrated that the common lipophilic pollutant BaP inhibited CD1a and CD1d expression on the surface of human DCs in a dose-dependent manner starting from a minimal inhibitory concentration detectable in exposed human populations^35^. We likewise established the inhibitory effect of BaP on the activation of lipid-specific T cells mediated by CD1a or CD1d proteins. The particulate form of PAHs also showed a marginal inhibitory effect on CD1a-mediated T cell activation. The transcriptomic analyses of BaP-exposed human DCs comprehensively display altered expression profiles for those genes involved in lipid metabolism, cytokine reactivity, and particularly endocytic function. To understand the impact of BaP on endocytic CD1 trafficking, we used imaging flow cytometry to demonstrate that the retention of CD1a and CD1d proteins in early and late endocytic compartments, respectively, was elevated by cellular exposure to BaP. Thus, BaP disrupts the endocytic function and impairs CD1 lipid antigen presentation on DCs for T cell activation. Our data support that lipid antigen presentation inhibited by BaP and other PAHs likely contributes to exacerbated inflammatory responses in diseases.

## Results

### Benzo[α]pyrene (BaP) inhibits CD1a and CD1d surface expression on human dendritic cells (DCs)

**Figure 1:**
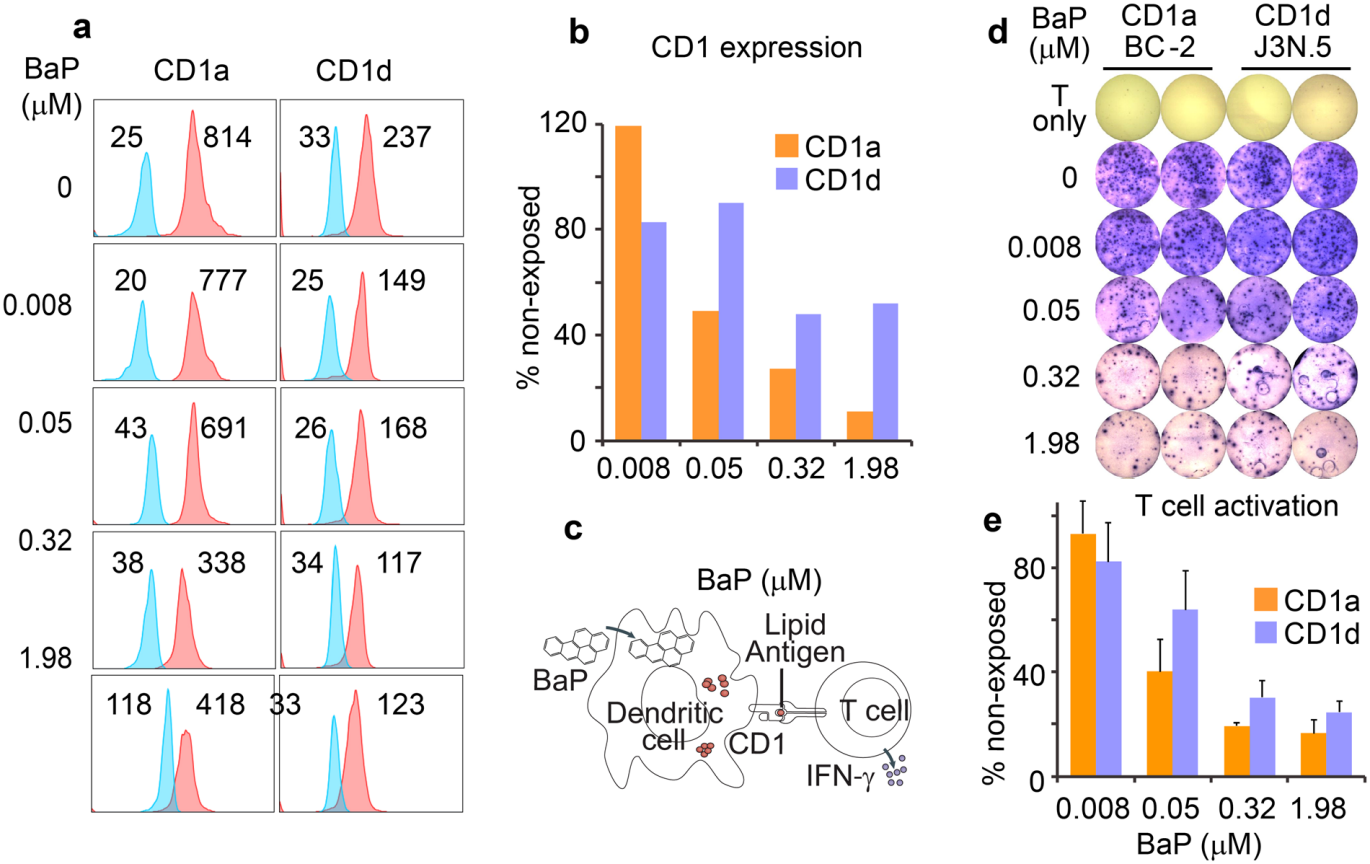
Inhibition of CD1 expression and T cell activation by BaP. CD1a and CD1d proteins (Red) on human DCs were tested by flow cytometry and plotted with non-stained controls that reflect the autofluorescence of BaP (Blue). To estimate CD1 expression, mean fluorescence intensity (MFI) was labeled by the histogram (**a**). Then, the MFI of stained samples was normalized to that of non-stained samples and compared with non-exposed DCs (**b**). T cell activation represented by IFN-γ production was stimulated by DCs and tested with an immunospot assay, as schematically shown (**c**). The immunospot plate (**d**) and quantified results (**e**) show that the activation of CD1a-restricted BC-2 cells and CD1d-restricted J3N.5 cells was inhibited in a dose-dependent manner by DCs exposed to BaP. Standard errors from technical replicates of each sample are shown. Data are from one experiment performed with blood cells obtained from an independent donor. Three experiments were performed with similar results.

Humans are commonly exposed to air pollution and chemically contaminated food as a consequence of an industrialized lifestyle and cigarette smoking^5, 35^. This environmental exposure releases harmful chemicals in human body fluids, including BaP and other PAH compounds. Particularly, BaP is commonly detectable in the blood and female follicular fluids of human populations who live in urban areas or smoke cigarettes^35, 36^. Since BaP aggravates multiple inflammatory diseases, full pathogenic elucidation and better therapy for these diseases are unlikely without understanding the human-environmental interactions in terms of how BaP alters T cell activation. In this study, we considered that the lipophilic nature of BaP likely contributes to its functional impact on lipid antigen presentation, which broadly involves lipid-protein interactions. We first tested the effect of BaP on altering the CD1 expression on human DCs, which were further used to stimulate lipid-specific T cells (Supplemental Fig. 1a). The surface expression of CD1 proteins was analyzed using flow cytometry as gated on the conventional DC population (Lineage^−^HLA-DR^+^CD11c^+^CD1c^+^) (Supplemental Fig. 1b). The result showed a reduction of the surface CD1a and CD1d expression in a dose-dependent manner for BaP exposure (Fig. 1a). The percentage of inhibition was calculated in comparison to a lack of exposure to BaP (Fig. 1b). Notably, the inhibitory effect began at very low BaP concentrations (8 nM and 50 nM), while an averaged and the highest BaP concentrations of 7.13 nM and 71.3 nM were detected in the human body fluids of smoker populations^35^. In this study, CD1b and CD1c expression was not included because these proteins were considered to be surface markers for gating conventional DCs^28^. Upon environmental exposure, BaP pollutants in air or food will cross epithelial barriers and enter blood circulation, subsequently coming into contact with various cell types including macrophages and DCs^4^. Our results demonstrate that lipophilic pollutant BaP sensitively alters the expression of CD1a and CD1d proteins on human DCs. Therefore, it is plausible that there is a direct impact from BaP and other PAH pollutants on human DCs in blood circulation and tissues, leading to a further understanding of functional outcomes and targeted pathways of pollutants *in vivo*.

### Inhibited activation of CD1a- and CD1d-restricted T cells

To determine whether the activation of lipid-specific T cells is also inhibited due to the reduced expression of CD1 proteins on human DCs, we co-cultured the BaP-exposed DCs with CD1a- and CD1d-restricted T cell lines. In this assay, DC cells were first exposed to different concentrations of BaP, and residual BaP was washed prior to co-culture with T cells. The activation of CD1a- and CD1d-restricted T cells was detected using immunospot assays by visualizing IFN-γ-producing spots (Fig. 1c). The reduced number of IFN-γ-producing spots from this assay reflects the inhibited activation of CD1a- and CD1d-restricted T cell lines in a dose-dependent manner for DC exposure to BaP (Fig. 1d). The percentage of T cell spots decreased with the increased exposure to BaP, demonstrating the dose-dependent effect of BaP on the activation of lipid-specific T cell subsets in humans (Fig. 1d and e). Similarly, the BaP concentrations with an inhibitory effect on CD1a- and CD1d-restricted T cells were detectable in human populations^35^, demonstrating a sensitive impact of BaP during a natural exposure condition in real life. Moreover, the BaP-mediated inhibitory effect appears more strongly on the endogenously generated lipid antigens (Fig. 1) rather than an exogenous ligand, such as the exogenously added α-galactosylceramide (Supplemental Fig. 1d). This result is consistent with the report in which the strong peptide antigen derived from ovalbumin can be loaded to the H-2K^b^ protein on the cell surface for the activation of CD8^+^ T cells, although the expression of H-2K^b^ was inhibited^37^. Since the impact of environmental pollutants on lipid antigen presentation in inflammatory diseases is generally mediated by endogenous lipid antigens, autoreactive lipid-specific T cells will be our focus in this study.

### Diesel exhaust particles (DEPs) inhibit CD1a expression and activation of the CD1a-restricted T cell line

**Figure 2:**
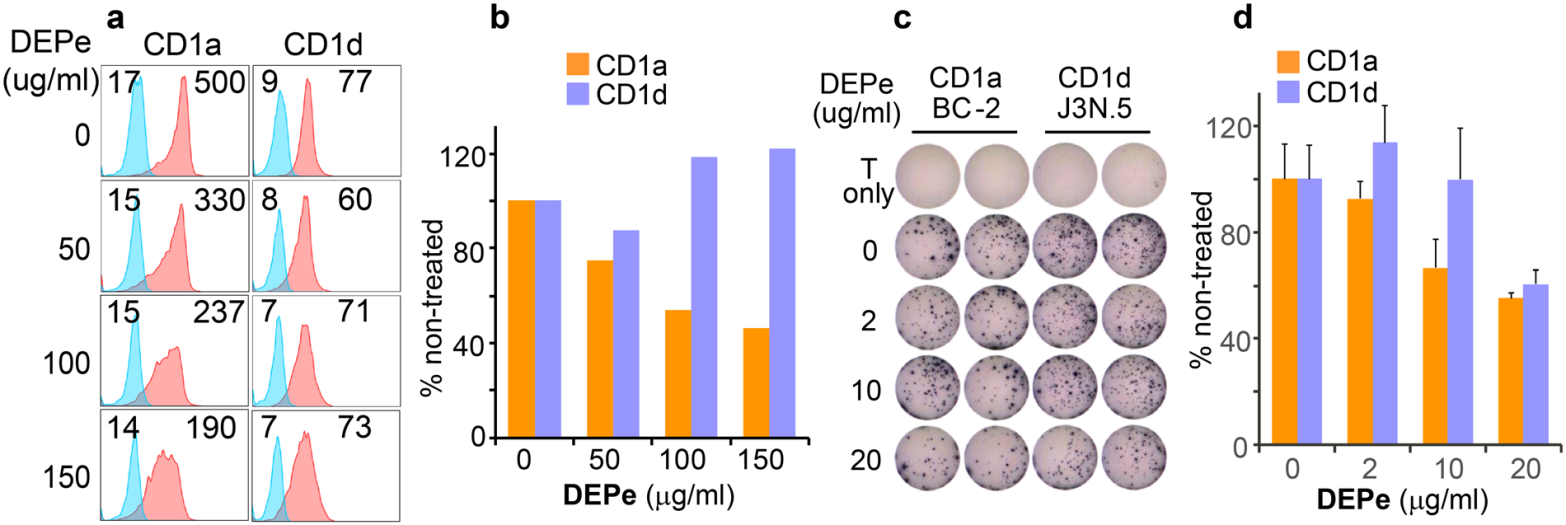
Inhibition of CD1a expression and T cell activation by DEPs. Expression of CD1a and CD1d proteins on DEP-exposed DCs and T cells responding to DEP-exposed DCs were similarly tested and shown as in Fig. 1. Mean fluorescence intensity (MFI) of CD1 staining was labeled by the histogram (**a**) and was also normalized to that of non-stained samples (**b**). T cell activation mediated by DEP-exposed DCs was tested by IFN-γ production and shown with an immunospot assay (**d**). Quantified immunospot results show certain inhibitory effect of DEP on the activation of CD1a-restricted BC-2 cells in a dose-dependent manner (**e**). Standard errors from technical replicates of each sample are shown. Data are from one experiment using cells from an independent donor. Two experiments were performed with similar results.

In addition to the vapor form (Supplemental Fig. 2a,b) of PAHs that transfers from blood to tissues and interact with DCs, the particulate form (Supplemental Fig. 2c) of PAHs also contributes to inflammatory responses and diseases, such as the exacerbation of asthma symptoms with an increased IL-17a level^38^. Diesel exhaust particles (DEPs) consist of agglomerates of primary carbon particles of around 100 nm in diameter with condensed hydrocarbons and PAH compounds that usually distribute as the droplets and liquid layer coating on the surface of carbon cores (Supplemental Fig. 2c)^39^. Upon inhalation, DEPs reach the alveolar and gas exchange regions of the lung, which contain different antigen-presenting cells^38^. The incubation of DEPs with DCs mimics the physiological interaction of DCs and T cells in the small airway tissues. Our results showed that the expression of the surface CD1a protein and the activation of CD1a-restricted T cells were inhibited by DEP-exposed DCs to a lesser degree than BaP-exposed DCs (Fig. 2), possibly due to lower BaP amounts in DEPs^39^. CD1d surface expression was not significantly impacted, although the activation of T cells restricted by CD1d-presented lipid antigens was marginally inhibited (Fig. 2). This result suggested that the particulate form of mixed PAHs potentially inhibits the activation of some subsets of lipid-specific T cells, indicating a harmful response in the lung tissue in addition to the exposure of blood and tissue DCs to free BaP as shown in Fig. 1.

### Gene expression profiles in DCs

**Figure 3:**
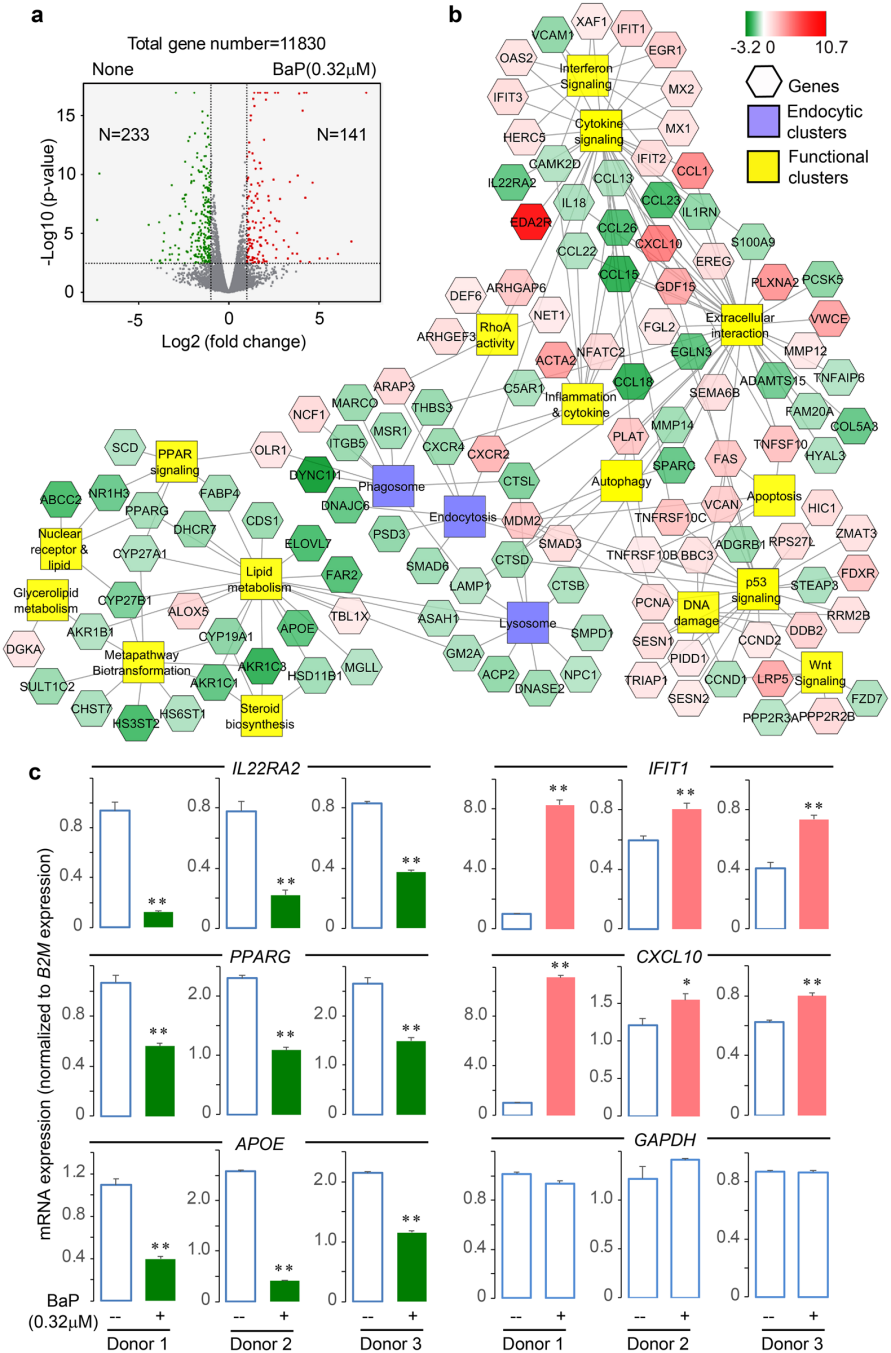
Altered gene expression profiles upon BaP exposure. Human monocyte-derived DCs from each donor (n = 3) were incubated with BaP and sorted for labeled conventional DCs, which were further used for RNA extraction and transcriptomic analysis. A volcano plot shows the number of differentially expressed genes (vertical lines = two-fold intensity difference, horizontal line = 0.05 false discovery rate-adjusted p-value) (**a**). The altered genes were clustered by functions (**b**). The expression level of some targeted genes was confirmed using RT-qPCR by normalization to *B2M* gene expression (**c**). Gene expression in BaP-exposed and non-exposed DCs was compared in three individual donors. The standard errors were calculated from triplicate reactions. The p-values were calculated using Student’s t-tests and are shown as **(p < 0.01) or *(p < 0.05).

We hypothesized that the overall inhibition of lipid antigen presentation by the lipophilic pollutants PAHs is mediated by the alteration of multiple processes, including lipid metabolism and endocytic lipid loading to CD1 proteins, which are critical for providing lipid antigens and maintaining CD1 surface expression^13, 15, 31–34^. To test this hypothesis and comprehensively determine the pathways targeted by BaP, we took a transcriptomic approach to profile the gene expression from human DCs upon BaP exposure. DCs were incubated with BaP at a concentration of 0.32 μM, which is around four times the highest reported concentration in humans^35^, to determine the altered gene profiles more comprehensively. This dose reflects an over-exposed condition to BaP or accumulative exposure to multiple PAHs and other lipophilic pollutants. We sorted the BaP-exposed human DCs (Lineage^−^HLA-DR^+^), the majority of which are conventional DCs (CD11c^+^CD1c^+^) (Supplemental Fig. 1b). After isolating polyA RNA from the sorted DCs and performing quality control on the isolated RNA, gene expression profiles were generated using the HiSeq sequencing platform. The differential expression between exposed and non-exposed groups underwent stringent statistical analyses using a p-value adjusted for the false discovery rate (FDRs) for triplicate samples. In this profiling analysis, we identified 141 and 233 genes with more than two-fold enhanced and reduced expression respectively at a significant p-value (<0.05), as listed in the supplemental Table 1 and shown in Fig. 3a. We further focused on pathway analyses using this altered gene pool, although the reported PAH receptor AHR^4^ was not found on this list, likely due to different expression kinetics or donor variability.

### Altered gene clusters involved in endocytic function and other pathways

**Figure 4:**
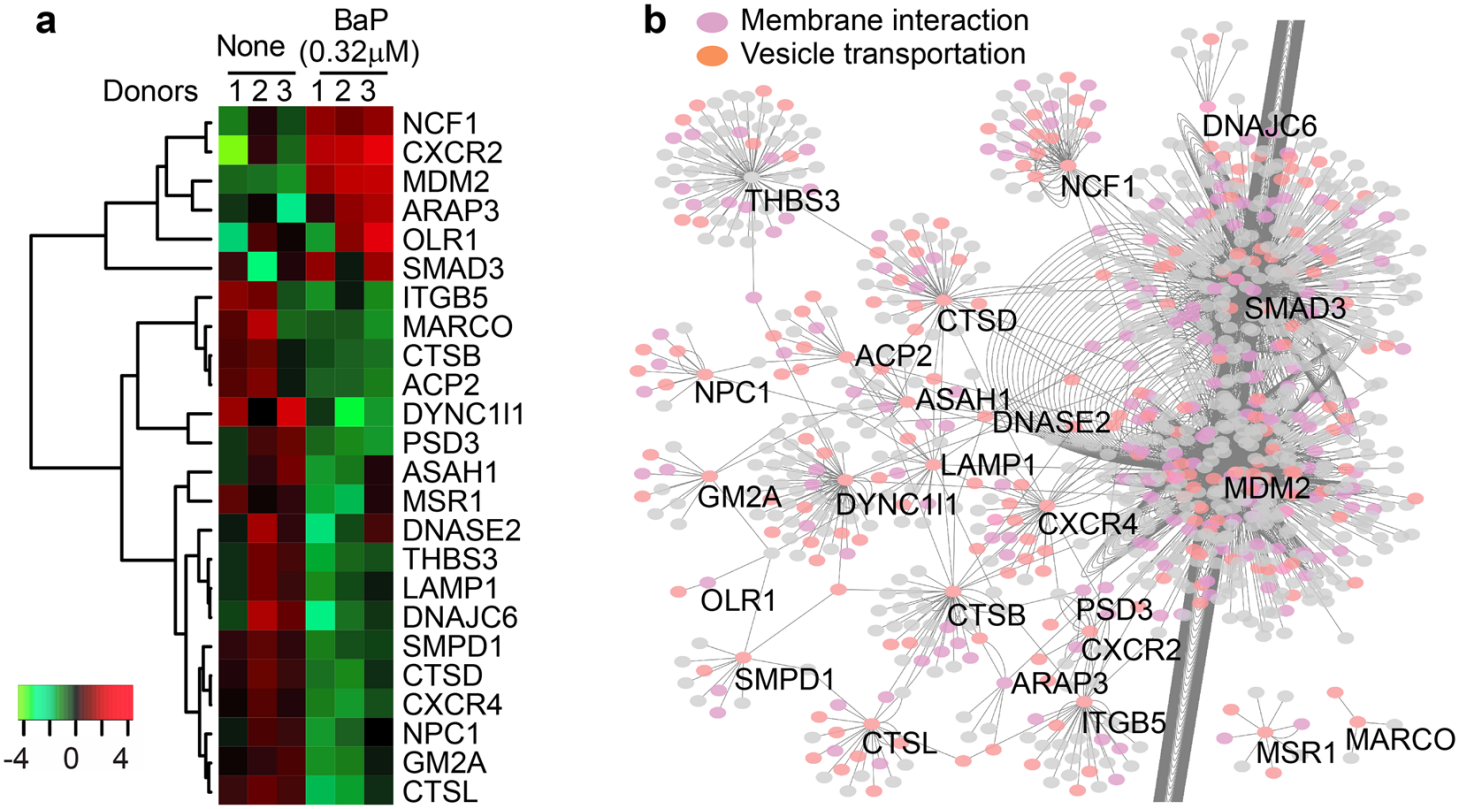
Gene expression and interaction of endocytic gene clusters. A heatmap shows the expression level of individual genes from endocytic clusters (**a**). The partner proteins interacting with these encoded proteins were predicted by searching against the protein interaction database BioGRID3.4 and visualized with Cytoscape. The result shows a putative interacting network highly relevant to membrane interactions and vesicle transportations (**b**). Each node represents a protein and each line represents an interaction.

To determine which cellular pathways are impacted by BaP and likely contribute to inhibited lipid antigen presentation, we used the ToppCluster software to obtain functionally enriched clusters from BaP-altered genes by searching multiple databases, including KEGG and REACTOME. When further visualized using the Cytoscape software, the altered gene clusters mostly involve lipid metabolism, xenobiotic transformation, endocytic function, cytokine reactivity, cell proliferation, and apoptosis (Fig. 3b). Several of these functional clusters may not be directly relevant to lipid antigen presentation, for example, the metapathway of biotransformation and Wnt signaling associates with xenobiotics metabolism or AHR-mediated responses^4^. The cytokine and chemokine responses usually impact T cell activation within the context of crosstalk between multiple cell types or in an *in vivo* setting. However, the functional clusters of the endocytic pathway and lipid metabolism appear to more directly regulate the lipid antigen presentation to T cells in this co-culture assay. In particular, the endocytic function has been intensively demonstrated to be important for lipid metabolite loading to CD1 proteins^26, 27^. Our findings show that BaP inhibits the expression of a number of genes involved in the function of endocytosis, phagosomes, and lysosomes (Fig. 3b, middle clusters). The expression level of some genes involved in cytokine signaling (*IL22RA2, IFIT1*, and *CXCL10*), and lipid metabolism (*PPARG* and *APOE*) was further confirmed with real-time quantitative reverse transcription PCR (RT-qPCR) in comparison to *B2M* expression (Fig. 3c) and *GAPDH* expression (Supplemental Fig. 3b), showing a high consistency of the BaP-altered expression level between transcriptomic and RT-qPCR analyses. In spite of donor variability in transcriptomic (Fig. 4a) or RT-qPCR analysis (Fig. 3c and Supplemental Fig. 3b), BaP-altered endocytic gene clusters are relevant to various biological functions, such as antigen loading to MHC class II or CD1 proteins (*CTSB, D, L*)^30^, lipid metabolism (*ACP2, ASAH1, SMPD1*), and lipid-receptor binding (*NPC1, GM2A, MSR1, OLR1*) (Fig. 3b and Supplemental Fig. 4), some of which have been shown to be involved in NKT cell development in mouse studies^40^. To further confirm the relevance to endocytic functions, we performed a protein interacting network analysis using the BioGRID database. The results with Cytoscape demonstrate that the partner proteins interacting with the protein products of BaP-altered endocytic genes broadly involve vesicle transportation and membrane function (Fig. 4b), further supporting that the endocytic pathway is one of the major pathways targeted by BaP exposure.

### Retention of the CD1a protein in early endocytic compartments

**Figure 5:**
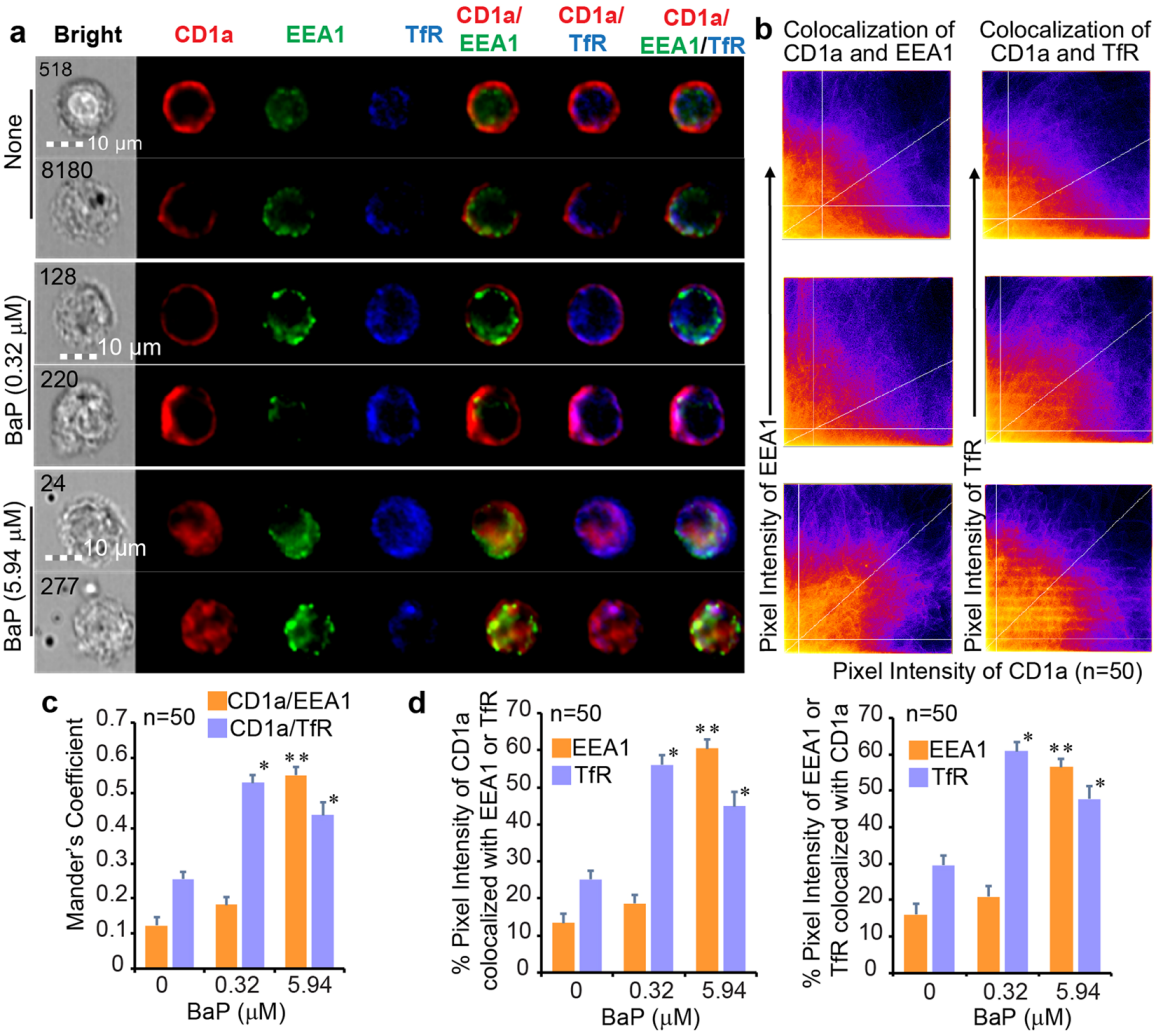
The CD1a protein retained in early and recycling endosomes. According to the gating strategy in Supplemental Fig. 5, two cellular images in the gated HLA-DR^+^CD11c^+^CD1a^+^EEA1^+^TfR^+^ population from each BaP-exposed condition are shown together with cell identities (numbers) and size bars (**a**). Fifty cells with strong co-stain and subcellular localization of CD1a, EEA1, and TfR proteins were extracted from the gated DC population from each of three BaP exposure conditions (none, 0.32 μM, and 5.94 μM), as exemplified in Supplemental Fig. 5b. Each cell image was manually input into ImageJ-Fiji and analyzed for the co-localization between CD1a and EEA1 proteins or between CD1a and TfR proteins. The co-localization of pixel intensity between different channels is first visualized with scatterplots (**b**), in which the horizontal and vertical lines represent Costes’s thresholds and the diagonal lines represent the ratio of overall pixel intensity between two channels. Moreover, co-localized areas were quantified with the Mander’s coefficient between CD1a and EEA1 proteins, and between CD1a and TfR proteins. Each column represents an averaged calculation of 50 cell images extracted from each of three BaP exposure conditions for one pair of co-localized proteins (**c**). Finally, the percentage of co-localized pixel intensity is also calculated. Similarly, each column represents an averaged calculation of multiple cell images (n = 50) from each BaP exposure condition for the annotated pair of co-localized proteins (**d**). Statistical significance (n = 50, p < 0.001) was obtained with Student’s t-tests in comparison to the non-exposed group (*) and lower exposure group (**) with indicated standard errors. Data are from one experiment using cells from an independent donor. Two experiments were performed with similar results.

CD1a binds a lipid antigen in early or recycling endosomes and CD1d captures lipid antigens in late endocytic pathways^26, 29^. Thus, subcellular localization of these two molecules will reflect the activities of different endocytic compartments. Specifically, retention of CD1a in early and CD1d in late endocytic compartments can be used to further functionally confirm altered endocytic gene profiles upon exposure to BaP. We first determined whether BaP impacts the function of early and recycling endosomes that control the lipid antigen loading to the CD1a protein^26, 29^. The early endosomes labeled with early endosome antigen 1 (EEA1) and the sorting or recycling endosomes labeled with transferrin receptor (TfR)^26, 29^ were co-stained with the CD1a protein and visualized using imaging flow cytometry. The obtained cellular profiles were gated using the IDEAS software and the cellular images of CD1a^+^HLA-DR^+^CD11c^+^ cells co-stained with either EEA1 or TfR proteins were extracted to visually show their staining intensity (Supplemental Fig. 5a and b). To understand the retention of CD1a in early endocytic compartments, we initially used bright detail similarity analysis in IDEAS to show a slight increase of co-localization between CD1a and EEA1 or TfR in some BaP-exposed conditions (Supplemental Fig. 5c). The bright detail similarity is calculated based on the Pearson’s correlation coefficient for testing the correlation of two factors, while our purpose is to understand the percentage of co-localized intensity and areas between CD1a and endocytic markers. However, the reproducible threshold, and percentage of co-localized areas and intensity cannot be calculated with IDEAS software and had to be analyzed with ImageJ-Fiji software^41^, which has also been used in several other studies^42–44^. Therefore, we extracted 50 cell images with a strong co-stain and a clear subcellular localization of CD1a, EEA1, and TfR proteins from the gated HLA-DR^+^CD11c^+^CD1a^+^EEA1^+^TfR^+^ cell population (Supplemental Fig. 5a and b). Two cell images from each BaP exposure condition are shown in Fig. 5a. Overall, the CD1a protein mostly expressed onto the cell surface and minimally distributed in early or recycling endosomes under the non-exposed condition (Fig. 5), supporting the normal function of lipid antigen loading and endosomal trafficking to transport the CD1a protein onto the cell surface. However, the CD1a protein significantly increased the percentage of co-localization with the TfR in recycling and sorting endosomes after BaP exposure (0.32 μM) (Fig. 5), and the endocytic gene expression was dramatically altered at this concentration (Figs 3 and 4). Furthermore, the CD1a protein accumulated in early endosomes upon exposure to a higher concentration of BaP (5.94 μM) (Fig. 5). The overlap of pixel intensity between EEA1 and CD1a or TfR and CD1a increased upon BaP exposure (Fig. 5b). The distribution of CD1a in early and recycling endosomes was also shown with an increased co-localized area tested by Mander’s coefficient (Fig. 5c) and an increased percentage of overlapped intensity (Fig. 5d). Student’s t-tests for the Mander’s coefficient and percent of overlapped intensity from each of the 50 cell images further demonstrated a statistical significance for the higher overlap of CD1a with EEA1 or TfR proteins in BaP-exposed DCs in comparison to non-exposed DCs (Fig. 5c and d). These observations strongly support that the CD1a protein begins to be retained in recycling endosomes at the low BaP concentration and is further retained in early endosomes at the high concentration, suggesting the contribution of altered endocytic gene expression to the reduced lipid antigen presentation, lower CD1a expression, and inhibited T cell activation. In addition, a similar analysis using a larger number of cell images (n = 100) was also performed to support that the co-localization analysis for the CD1a and early endocytic markers was consistent and reproducible (Supplemental Fig. 6).

### Retention of CD1d protein in late endocytic compartments upon BaP exposure

**Figure 6:**
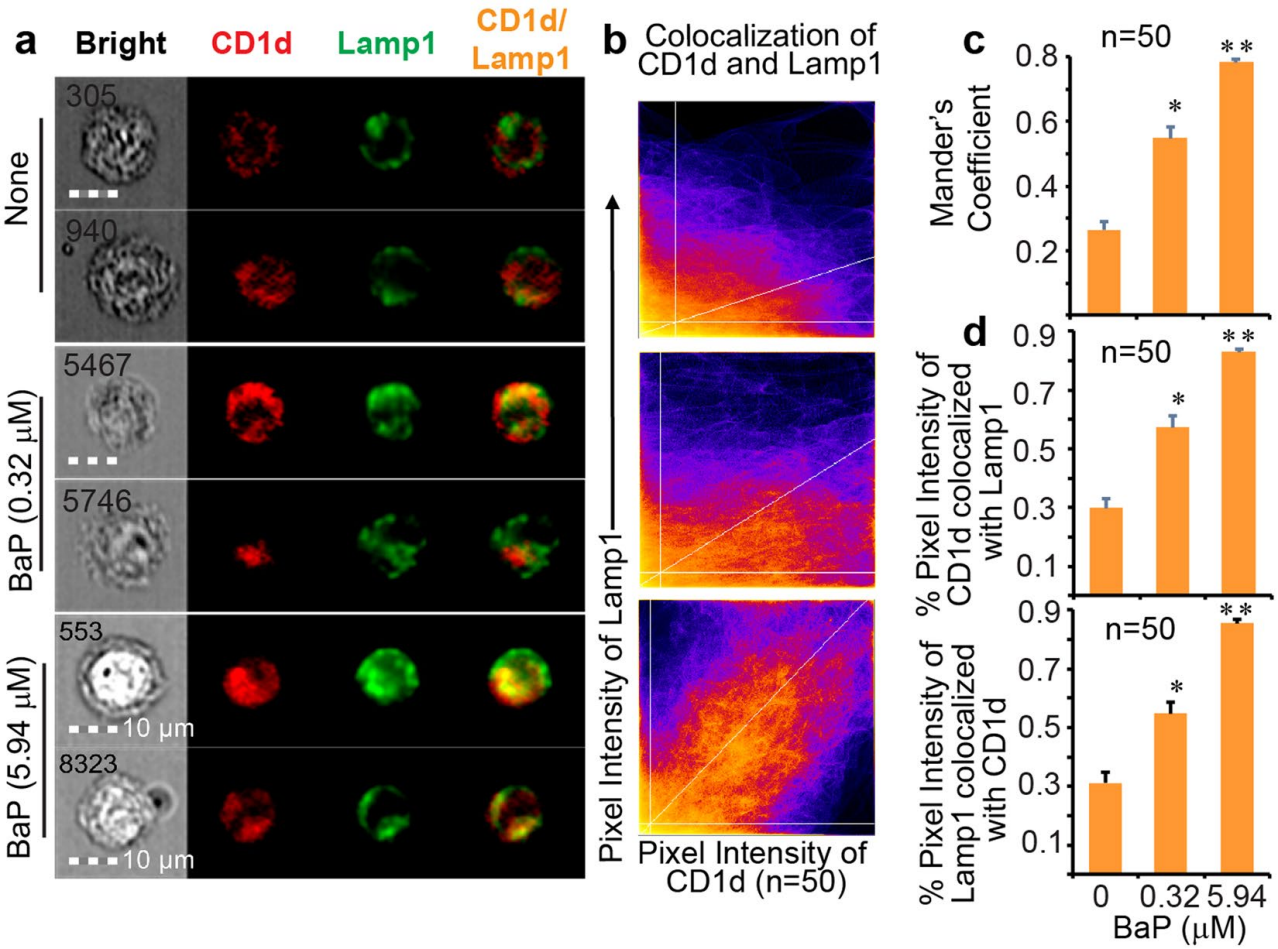
The CD1d protein retained in late endocytic compartments. Representative images of human DCs from the gated HLA-DR^+^CD11c^+^CD1d^+^Lamp1^+^ cells are shown together with cell identities and size bars (**a**). Fifty cells with strong co-stain and subcellular localization of CD1d and Lamp1 proteins were extracted from the gated DC population from each of three BaP exposure conditions (none, 0.32 μM, and 5.94 μM). In this ImageJ-Fiji analysis, the co-localized pixel intensity between CD1d and Lamp1 proteins is first shown with scatterplots (**b**). In parallel to that in Fig. 5, Mander’s coefficients were further calculated and are shown using each column to represent an average calculation of 50 cell images extracted from each of three BaP exposure conditions (**c**). The percentage of co-localized pixel intensity was also calculated and is similarly shown (**d**). Statistical significance (n = 50, p < 0.001) was obtained with Student’s t-tests in comparison to the non-exposed group (*) and lower exposure group (**) with indicated standard errors. Data are from one experiment using cells from an independent donor. Two experiments were performed with similar results.

Complementary to CD1a in endocytic trafficking, CD1d loads lipid antigens in late endosomes and lysosomes^26^. This feature allows us to comprehensively determine the impact of BaP on both late endocytic compartments, reflected by subcellular localization of the CD1d protein, and early endosomal compartments, reflected by localization of the CD1a protein^29^. Similarly, to measure the co-localization of CD1d with the late endocytic marker protein, lysosomal-associated membrane protein 1 (Lamp1), we gated the HLA-DR^+^CD11c^+^CD1d^+^Lamp1^+^ cell population using IDEAS and extracted 50 cell images, based on the technical criteria of a strong co-stain between CD1d and Lamp1 proteins, and a subcellular localization of staining signals, for further imaging analysis using Fiji in the ImageJ package. The results showed that the CD1d protein was mostly separated from Lamp1 in the non-exposed condition. Moreover, CD1d-Lamp1 co-localization increased as a dose-dependent effect of BaP exposure, based on cell images (Fig. 6a), overlapped pixel intensity at diagonal areas (Fig. 6b), enhanced Mander’s coefficients (Fig. 6c), and increased percentage of overlapped intensity (Fig. 6d). These analyses demonstrate the intracellular retention of CD1d protein in the late endocytic pathway upon cellular exposure to BaP, supporting reduced CD1d surface expression, inhibited CD1d-restricted T cell activation, and altered endocytic gene expression parallel to the inhibited CD1a-mediated lipid antigen presentation. Similarly, the co-localization of CD1d and Lamp1 proteins was confirmed using a larger number of cell images (n = 100), additionally supporting the consistency and reproducibility of the co-localization analysis (Supplemental Fig. 7).

## Discussion

Lipid antigen presentation depends on a series of processes, including lipid metabolism, extracellular lipid transportation, intracellular lipid transfer, lipid binding to CD1 proteins, CD1 trafficking to the cell surface, and interaction of the CD1-lipid complex with T cell receptors, all of which feature intensive lipid-protein interactions^13, 17, 45^. The underlying mechanism for the inhibition of lipid antigen presentation by BaP exposure can likely be attributed to interference with these processes. First, lipid metabolism is critical in providing lipid metabolites to be bound and presented by CD1 proteins. Lipidomic analyses have shown that human CD1 proteins bind a broad range of lipid metabolites, including sphingolipids, glycosphingolipids, lyso- and ether-phospholipids, acylglycerols, wax esters, and fatty acids^15, 17, 31–33^. For example, skin-derived squalene, a metabolite from cholesterol metabolism^46^, was identified as an antigen presented by the CD1a protein for T cell activation, which facilitates cytokine secretion to regulate inflammatory responses^15, 16^. CD1-associated lyso- and ether-phospholipids, related to the production of fatty acids and peroxisomal synthesis of ether lipids, are crucial for the development and activation of NKT cells^47, 48^. Therefore, production of CD1-associated lipids as metabolites generated from various lipid metabolic pathways in differential intracellular compartments^49^ is critical for lipid antigen presentation by CD1 proteins. If BaP and other PAHs interfere with lipid metabolisms, these pollutants will alter the supply of cellular lipid metabolites to be presented by CD1 proteins. Indeed, exposure to BaP decreases the production of free fatty acids but increases fat mass in human and mouse adipocytes^7^. PAH exposure also increases lipid peroxidation products in the blood of exposed animals^50^. Our transcriptomic data from BaP-exposed DCs show altered expression of a number of lipid metabolic enzymes (Figs 3 and 4), potentially resulting in altered lipid metabolites for CD1 binding. Second, in addition to altered gene expression in lipid metabolism (Fig. 3b and c, and Supplemental Fig. 3b), the results also showed reduced gene expression of lipoproteins (e.g., apolipoprotein E [APOE]) and lipid transfer proteins (e.g., fatty acid binding protein 4 [FABP4]) as well as inhibited surface expression of CD1 proteins (Figs 1, 2, 3 and 4). Thus, some proteins involving extracellular lipid transportation and intracellular lipid transfer are altered. These data suggest that multiple steps in the lipid antigen presentation pathway are susceptible to BaP, through downstream perturbation of gene expression upon BaP binding to xenobiotic receptors^4^, direct hydrophobic disruption of lipid association with diverse proteins, or both. As a result, the accumulated effects likely influence multiple processes in the lipid antigen presentation pathway, leading to inhibited CD1 surface expression and T cell activation. Therefore, future studies on lipid metabolism and lipid metabolite loading to CD1 proteins using our lipidomic platform^15, 33, 51^ will provide a deeper molecular understanding of the perturbation effect mediated by BaP and other lipophilic pollutants.

To further support the notion that BaP interferes with multiple processes contributing to lipid antigen presentation, we demonstrated blockage of CD1a and CD1d trafficking from endocytic compartments to the cell surface by BaP (Figs 5 and 6) together with evidence of altered expression of endocytic gene clusters. The activation of lipid-specific T cells has been shown to rely on antigen loading through the endocytic pathway, which is similarly used by MHC class II proteins to activate conventional CD4^+^ T cells^26, 52^. The BaP-altered endocytic gene clusters, including endocytosis, phagosome, and lysosome clusters (Figs 3 and 4), indicate that endocytic lipid antigen loading and CD1 trafficking to the cell surface are likely impaired. Although the dependence of lipid antigen presentation on the endocytic pathway is well known, we showed in this study that BaP independently altered the gene expression profiles involved in endocytic lipid metabolism and membrane trafficking. This transcriptomic observation is consistent with the reduced surface CD1 expression and inhibited T cell activation by BaP exposure (Fig. 1). From a different perspective, environmental pollutants are alternative tools for dissecting the host elements that contribute to functional lipid antigen presentation for T cell activation. Indeed, CD1a trafficking from early and recycling endosomes to the cell surface, and CD1d trafficking from late endocytic compartments to the cell surface are blocked in human DCs upon BaP exposure (Figs 5 and 6).

In the current industrial age, environmental pollutants are important risk factors for inducing or worsening inflammatory diseases. The vapor form of PAHs, when inhaled by humans, will enter the airway epithelia and blood circulation to contact various cell types, including macrophages and DCs in blood and tissues^53^. The particulate form of PAHs, such as DEPs, can either interact with macrophages, DCs, and other immune cells in the lung tissues or chemically dissolve and transfer through blood circulation^38, 39^. Transfer of BaP from lung to distal tissues, including various lympoid tissues, has been supported by previously tested BaP blood concentrations in human populations^35^. The toxic doses of BaP in this study are comparable to its detectable concentrations in human populations, reflecting the critical role of BaP in inducing or exacerbating human inflammatory diseases and cancers in an unnoticeable manner. Based on their lipophilic chemical nature, PAHs are considered prone to interact with the abundant lipid compositions of cells and the hydrophobic domains of some proteins, as evidenced by or predicted in previous studies^10, 12^. The functional correlation between lipophilic pollutants and inflammatory diseases can be further established through the lipid antigen presentation pathways, because T cells activated by lipid antigens are important anti-inflammatory or regulatory cells in inflammatory diseases^18, 54^. Specifically, we speculate that this inhibitory effect of BaP and other PAHs will minimize the regulatory functions of lipid-specific T cells. These regulatory functions have been reported in obese individuals and animals^20^ as well as in other inflammatory disorders, including lupus, lung inflammation, preterm birth, and autoimmune diseases^55, 56^. Therefore, the reduced expression of surface CD1 proteins on peripheral immune cells and the inhibited responses of lipid-specific T cells upon BaP exposure are an interesting avenue for future research into whether these are candidate biomarkers for assessment of the impact of lipophilic pollutants in human populations and diseases.

In summary, our data demonstrate that the common lipophilic pollutant BaP sensitively inhibits lipid antigen presentation and T cell activation mediated by CD1a and CD1d proteins by altering the gene expression and function of endocytic compartments. The particulate form of PAHs also shows an inhibitory effect on CD1a lipid antigen presentation. Our findings provide strong evidence to support a previously unrevealed human-environmental interaction pathway by which lipophilic pollutants target lipid antigen presentation, potentially exacerbating inflammatory responses in various diseases. Based on the currently known functions of lipid-specific T cells in various inflammatory diseases^18, 19^ and the high frequency of these T cells in responding to self-lipids^14, 15^, we believe that lipid antigen presentation will provide a new functional and biochemical link between exposure to lipophilic environmental pollutants and T-cell-regulated inflammatory diseases. Ultimately, cumulative findings from these studies can provide targets for designing novel biomarkers and therapies for the inflammatory diseases and cancers that are exacerbated by environmental pollutants.

## Materials and Methods

### Chemicals of environmental pollutants

Benzo[a]pyrene (Alfa Aesar, Cat# 15856, crystalline with a purity of 96%) was dissolved in dimethyl sulfoxide (DMSO) at the concentration of 10 mg/ml as a stock and stored at −80 °C. The BaP stock was further serially diluted using PBS or culture medium to various concentrations including the lowest concentration of 8 nM.

### Antibodies for flow cytometry staining on human DCs

If not specifically indicated, antibodies used in this study were purchased from Biolegend. Biotinylated anti-CD3 (clone UCHT1), CD19 (HIB19), CD20 (2H7), CD14 (M5E2), and CD56 (HCD56) were used for lineage label of the non-dendritic cells in monocyte culture. APC/Cy7-labeled anti-HLA-DR (L243), PE/Cy5-anti-CD11c (Bu15), PE/Cy7-anti-CD303 or BDCA-2 (201 A), Alexa Fluor 647-anti-CD1b (1B8), Brilliant Violet 421-anti-CD1c (L161), and PE-anti-CD1d (51.1) were used for labeling human dendritic cells as reported^28^. Anti-CD1a (OKT6) was a gift from Branch Moody at Brigham & Women’s Hospital or purified from ATCC cell line. Purified anti-CD1a was labeled with FITC or Pacific Blue using antibody labeling kits (Invitrogen).

### Differentiation and flow cytometry assay of human peripheral DCs

The human protocols in this study were approved by the Institutional Review Board of University of Cincinnati, including obtaining written informed consents from all subjects, and all methods were performed in accordance with the relevant guidelines and regulations. Blood samples from healthy donors free of detectable infectious or non-infectious diseases were obtained from the Hoxworth Blood Center at University of Cincinnati Medical Center. In this study, blood cells from each independent donor were used to perform one experiment and each conclusion was drawn based on similar results from two or three experiments. Since we investigated the BaP-altered DC function for T cell activation, exposed and non-exposed conditions were compared using blood cells from the same donors. Thus, the biological and technical variation was minimal. As in Supplemental Fig. 1, peripheral blood mononuclear cells (PBMCs) were isolated using Ficoll-paque gradient (GE Healthcare). Modified from the reported approach^28^, the isolated PBMC were treated with 300 U/ml human recombinant GM-CSF (Biolegend, San Diego, CA) for 30 mins and the adhered cells were further stimulated with human recombinant GM-CSF (300 U/ml) and IL-4 (200 U/ml) for 5 days (Supplemental Fig. 1). Twenty four hours prior to flow cytometry assay, lipopolysaccharide (LPS) purchased from Sigma (L6143) was added at 50 ng/ml to activate DCs. For an aliquot of 0.1 million DCs, the surface staining of CD1 proteins and markers were performed upon sufficient blockage of Fc receptors with combination of at least 2 ug of each anti-human Fc receptor antibody, including anti-CD16, CD32, and CD64 antibodies, together with over-dosed human serum blocker FcX (Biolegend). This blockage assured that the staining with isotype control antibodies was similar to the non-stained samples (Supplemental Fig. 1c). Then, the standard staining protocol was followed. Flow cytometry assay was performed using Millipore Guava EasyCyte 12 channel high throughput flow cytometer in the lab according to the manufacturer’s instruction. Flow cytometry data were analyzed with Millipore Guava InCyte and Flowjo software (Supplemental Fig. 1).

### ELISPOT assay for the activation of lipid-specific T cells

BaP-pulsed DCs were harvested and washed to remove residual BaP composition in culture media to avoid the direct impact of BaP on T cells. These BaP-pulsed DCs were confirmed with the survival rate of 80% or higher using trypan blue-staining prior to T cell activation assays. Then, twenty thousand human DCs and three thousand CD1a-restricted T cell line BC-2^15^ or CD1d-restricted T cell line J3N.5^57^ were co-incubated for 16 h on a multiscreen filter plate (Millipore) coated with anti-human IFN-γ antibody (Mabtech) according to the manufacturer’s instructions. The IFN-γ-secreting spots were shown with the sequential staining steps using the biotinylated anti-human IFN-γ antibody (Mabtech), ExtraAvidin alkaline phosphatase (Sigma), and substrate BCIP/NBT (Sigma). The resulted staining was visualized and quantified using CTL-ImmunoSpot S6 Micro Analyzer.

### RNA sequencing

Human DCs were sorted for Lineage^−^HLA-DR^+^ cells that enrich with conventional DCs. Around ten thousand cells were collected and dissolved in lysis solution for preparing total RNA using mirVana kit (ThermoFisher). For directional RNA sequencing, about 200 ng total RNA was input for constructing mRNA library using PrepX mRNA Library preparation kit (WaferGen) and Apollo 324 NGS automated library preparation platform (WaferGen). Briefly, the purified mRNA from high quality total RNA was confirmed with Agilent bioanalyzer and subjected to automated RNase III fragmentation, adaptor ligation, and cDNA synthesis, followed by Agencourt AMPure XP beads (Beckman) purification. Using universal and index-specific primers with limited PCR cycle number of 13, sample-specific index was added to each ligated cDNA and the amplified library was enriched by AMPure XP beads purification with the final elution volume of 16 µl. To estimate the quality and yield of the purified library, one µl of library sample was analyzed using DNA high sensitivity chip (Agilent). To accurately quantify the library concentration for the clustering, the library was 1:10^4^ diluted in dilution buffer (10 mM Tris-HCl, pH 8.0 with 0.05% Tween 20), and qPCR-measured by Kapa Library Quantification kit (Kapabiosystem) using ABI’s 9700HT real-time PCR assay (Lifetech). Individually indexed libraries were proportionally pooled (~25 million reads per sample) for clustering in cBot assay (Illumina). Libraries at the final concentration of 15.0 pM was clustered onto a flow cell using Illumina’s TruSeq SR Cluster kit v3, and sequenced for 50 cycles using TruSeq SBS kit on Illumina HiSeq sequencing assay.

### Bioinformatic and statistical analysis of altered gene clusters

Sequence reads were aligned to the genome and converted to intensity counts. These resultant gene expression intensity counts were compared between BaP-exposed and non-exposed DCs from three donors, using the edgeR Bioconductor package. Differentially expressed genes between the exposed and non-exposed DCs were then identified based on the absolute fold change (>2 folds) and the false discovery rates (FDRs)-adjusted p-values (<0.05). To predict the functional clusters of differentially expressed genes, we used ToppCluster software package to search these genes against several databases including KEGG and REACTOME and generate clustering data. ToppCluster uses the hypergeometric test to obtain functional enrichment achieved via the gene list enrichment analysis (https://toppcluster.cchmc.org/). The results from these function clusters were further input to software Cytoscape Version 3.3.0 (www.cytoscape.org/), a broadly used open source software platform for visualizing complex networks. Thus, the genes involved in different clusters or pathways, including endocytic clusters, lipid metabolism, cell proliferation, and signaling pathways etc. are shown in a network with color annotation of the averaged fold change of gene expression. For the genes involved in endocytic pathways, the partner proteins that interact with these differentially expressed genes were also predicted using protein interaction database BioGRID3.4 (http://thebiogrid.org/), which searches 56,621 publications for 1,066,335 protein and genetic interactions from major organisms. The list of interacting partner proteins was input to Cytoscape for network visualization.

**RT-qPCR** was performed with the SuperScript III kit (Thermofisher) using ~100 ng total RNA extract that was used in previous RNA sequencing analysis. Primers for real-time RT-qPCR were designed via NCBI Primer-BLAST tool (Supplemental Fig. 4a) and tested for their specific amplification in agarose gel. The expression level of the targeted mRNA was analyzed in triplicate using Power SYBR Green Master Mix (Thermofisher) and 7900HT Fast Real-Time PCR instrument (Thermofisher). The PCR condition was 50 °C 2 min, 95 °C 10 min, followed by 40 cycles of 95 °C 15 sec, 56 °C and 68 °C 60 sec. The relative level of gene expression was normalized by housekeeping genes *GAPDH* and *B2M*, and calculated by the 2^−ΔΔCt^ method [PMID: 18560533].

### Imaging flow cytometry

Human dendritic cells exposed to BaP at the concentrations of 0.32 μM and 5.94 μM, and at non-exposed condition were first blocked with anti-human Fc receptor antibodies and human serum blocker similar to regular flow cytometry staining. The pacific blue-labeled anti-CD1a (OKT-6, ATCC), PE/Cy7-anti-HLA-DR, and PE/Cy5-anti-CD11c was used to first perform the surface staining. Then the DCs were sequentially fixed with 4% paraformaldehyde in PBS and permeabilized with Permeabilization Wash Buffer (Biolegend). The intracellular staining was performed with a mixture of Pacific blue-labeled anti-CD1a, FITC-anti-EEA-1 (14/EEA1, BD Transduction Laboratories), and APC/H7-anti-transferrin receptor or CD71 (M-A712, BD Pharmigen). For CD1d protein, the purified anti-human CD1d (51.1) was used for both surface and intracellular staining. The Alexa fluor 647-labeled anti-Lamp1 (CD107a) (H4A3) was used for intracellular staining. The stained samples were analyzed using Millipore ImageStream X imaging flow cytometer at the flow cytometery core of Cincinnati Children’s Hospital following the manufacturer’s instruction. We used 40X objective to yield an approximate pixel size of 0.5 μm by 0.5 μm (1 pixel) of the object. The imaging flow cytometry allows acquisition and analysis of a substantial number of cellular images in an unbiased manner.

### Co-localization analyses of cell images

The acquired cellular images were first analyzed using IDEAS 6.2 to perform compensation using single-stained versus non-stained samples and to normalize BaP autofluorescence. The stained cells were then gated to obtain the subsets of HLA-DR^+^CD11c^+^CD1a^+^EEA1^+^TfR^+^ (Supplemental Fig. 5a,b, and c) and HLA-DR^+^CD11c^+^CD1d^+^Lamp1^+^ cells. To determine whether BaP exposure impacts the endocytic trafficking of CD1a and CD1d proteins, we further test the co-localization between CD1a and EEA1, CD1a and TfR, or CD1d and Lamp1 molecules at different BaP concentrations. First, IDEAS was used to sensitively gate the cells with invisible, minimal, or excessive staining signals (Supplemental Fig. 5a and b) and perform a Pearson’s-correlation-based calculation (subsequently plotted as a bright detail similarity). This calculation is to show the correlation of co-stained areas with a manually set intensity threshold for hundreds of gated cell images (Supplemental Fig. 5c). However, the co-localization analyses based on pixelated staining intensity, reproducible threshold setting, and percentage of co-localized areas or intensity could not be assessed with the bright detail similarity analysis in IDEAS. In contrast to this disadvantage in IDEAS analysis, ImageJ-Fiji is advantageous in performing aforementioned analyses in a statistically significant manner, similar to our previous co-localization analyses for confocal images^58^. Thus, we further extracted cell images only based on two technical inclusion criteria, the visual presence of strong co-stained signals and subcellular localization, for the co-localization analyses of CD1 proteins with endocytic markers using ImageJ-Fiji. Fifty cell images with CD1a, EEA1, and TfR staining, and fifty cell images with CD1d and Lamp1 staining were extracted from non-exposed, low-dose-BaP-exposed, and high-dose-BaP-exposed human DCs, respectively (Supplemental Fig. 5a and b). In ImageJ-Fiji analysis, we used a scatterplot to visualize the co-localization of the pixel intensities between CD1a (Red) and EEA1 (Green), CD1a (Red) and TfR (Blue), or CD1d (Red) and Lamp1 (Green) channels (Figs 5b and 6b). The auto threshold determination was performed with Costes’s method and shown as white lines parallel to axes. Costes’s threshold determination is able to successfully distinguish true overlap as low as 3% from a random color pixel, as verified by fluorescence resonance energy transfer^59^. Only the intensity levels above the threshold for both channels were considered for further co-localization analyses. A linear regression was generated and shown with a diagonal white line, the gradient of which is the ratio of the intensities from two channels. This method is fully reproducible, with the same resulted thresholds for the same data sets and similar thresholds for similar datasets. The Mander’s split co-localization coefficients (0 means no co-localization and 1 means perfect co-localization) were calculated and averaged from the measurement of multiple cell images (n = 50 or n = 100) from each of three BaP exposure conditions to show the proportion of the thresholded intensity signal in one channel that co-localizes with the other channel (Figs 5c and 6c). Similarly, ImageJ-Fiji was also used to calculate the percent of thresholded pixel intensity co-localized between CD1 and endocytic markers for multiple cell images (n = 50 or n = 100) from each of three BaP exposure conditions, including non-exposure, low-dose exposure, and high-dose exposure, respectively (Figs 5d and 6d).

### Statistical analyses of co-localized pixel intensity

Student’s t-tests were then performed to test the significant difference for the Mander’s coefficient between BaP-exposed versus non-exposed or high dose BaP versus low dose BaP conditions (n = 50 in Figs 5c and 6c, and n = 100 in Supplemental Figs 6c and 7c). Similarly, Student’s t-tests were also applied to test the significant difference of the percent thresholded pixel intensity for co-localized CD1a and EEA1, CD1a and TfR, or CD1d and Lamp1 proteins between BaP-exposed versus non-exposed or high dose BaP versus low dose BaP conditions (n = 50 in Figs 5d and 6d, and n = 100 in Supplemental Figs 6d and 7d).

## Electronic supplementary material

Supplemental figures and table
