# Supplementary material for: Inhibition of endocytic lipid antigen presentation by common lipophilic environmental pollutants
Source: Sci Rep. 2017 May 18;7:2085. doi: 10.1038/s41598-017-02229-7 (PMC5437007; doi:10.1038/s41598-017-02229-7)
Supplement: Supplementary file 1 — Supplemental figures and table [file 41598_2017_2229_MOESM1_ESM.pdf]

Inhibition of endocytic lipid antigen presentation by common lipophilic environmental pollutants

Manju Sharma[1], Xiang Zhang[2], Shuangmin Zhang[1], Liang Niu[3], Shuk-mei Ho[1], Aimin Chen[4], Shouxiong Huang[1,5]*

[1]Division of Environmental Genetics and Molecular Toxicology,

[2]Genomics, Epigenomics and Sequencing Core,

[3]Division of Biostatistics and Bioinformatics,

[4]Division of Epidemiology,

[1,2,3,4]Department of Environmental Health, University of Cincinnati College of Medicine, Cincinnati, OH 45267

*Correspondence to Shouxiong Huang: Shouxiong.huang@uc.edu

Supplementary Information

Seven supplemental figures and one supplemental table are included.

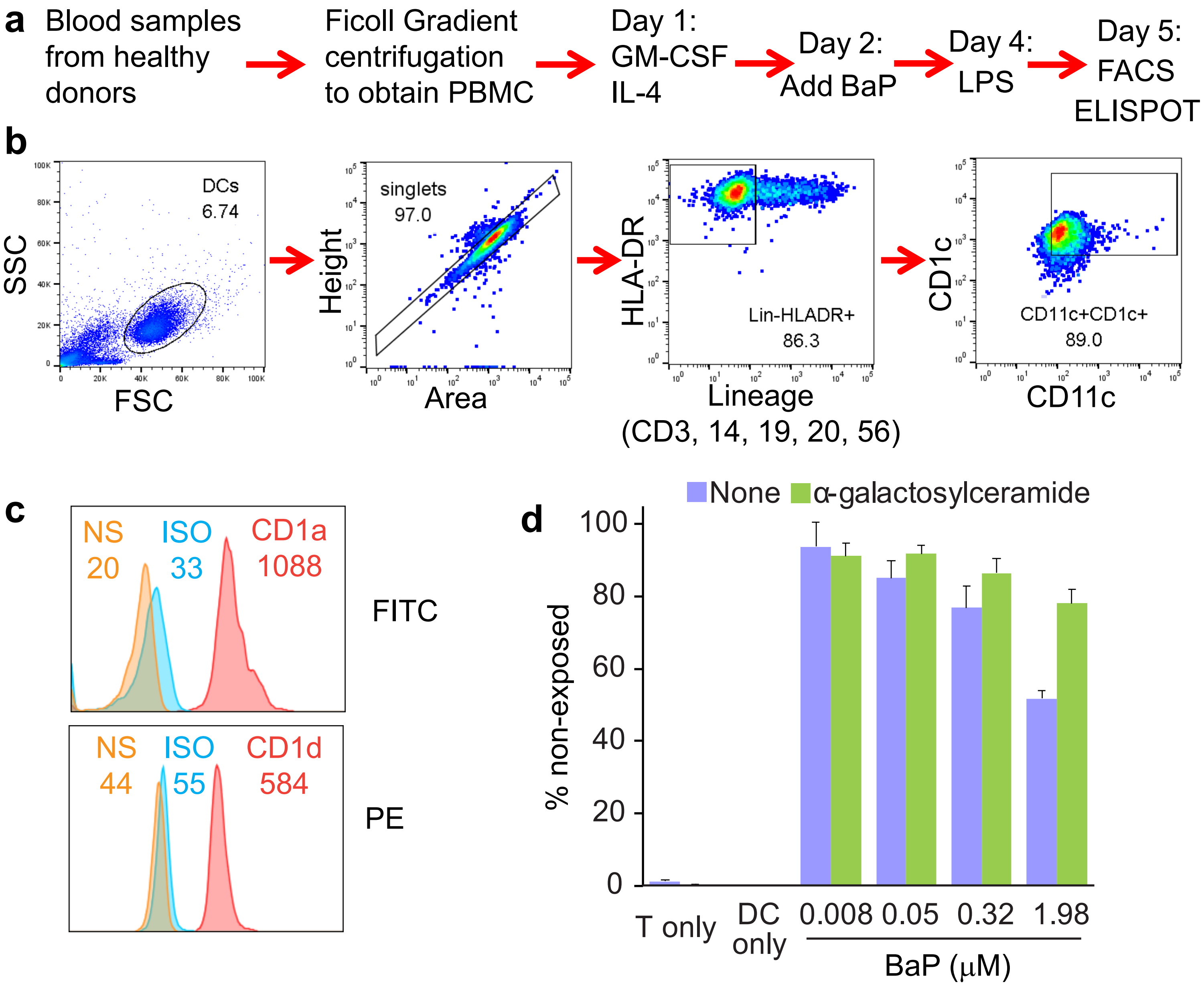

Supplemental Figure 1. Generation and gating of monocyte-derived dendritic cells, and impact of BaP exposure on α–galactosylceramide presentation. Monocyte-derived DCs were differentiated using cytokines granulocyte macrophage colony-stimulating factor (GM-CSF, 300 U/ml) and interleukin-4 (IL-4, 200 U/ml), and activated using lipopolysaccharide (LPS, 50 ng/ml). PBMC: peripheral blood mononuclear cell. (a). Conventional dendritic cells (DCs) were gated on Lineage^{-}HLA-DR^{+}CD11c^{+}CD1c^{+} population (b). Fc receptor blocking antibodies efficiently blocked the background staining shown by isotype-matched irrelevant antibodies (ISO, blue) in comparison to non-stained (NS, yellow) and CD1 antibody staining (red) with indicated mean fluorescence intensity (c). Finally, only minimal impact of BaP exosure on the presentation of α–galactosylceramide in DCs for NKT cell activation (J3N.5 cell line, green) was shown in comparison to the stronger impact on the presentation of endogenous lipid antigens (purple) using an ELISPOT assay (d). Error bars indicate the standard errors of triplicate samples from one donor and the data represent the independent experiments from two donors with similar results. DC only and T only represent control groups without DC and T cell co-culture.

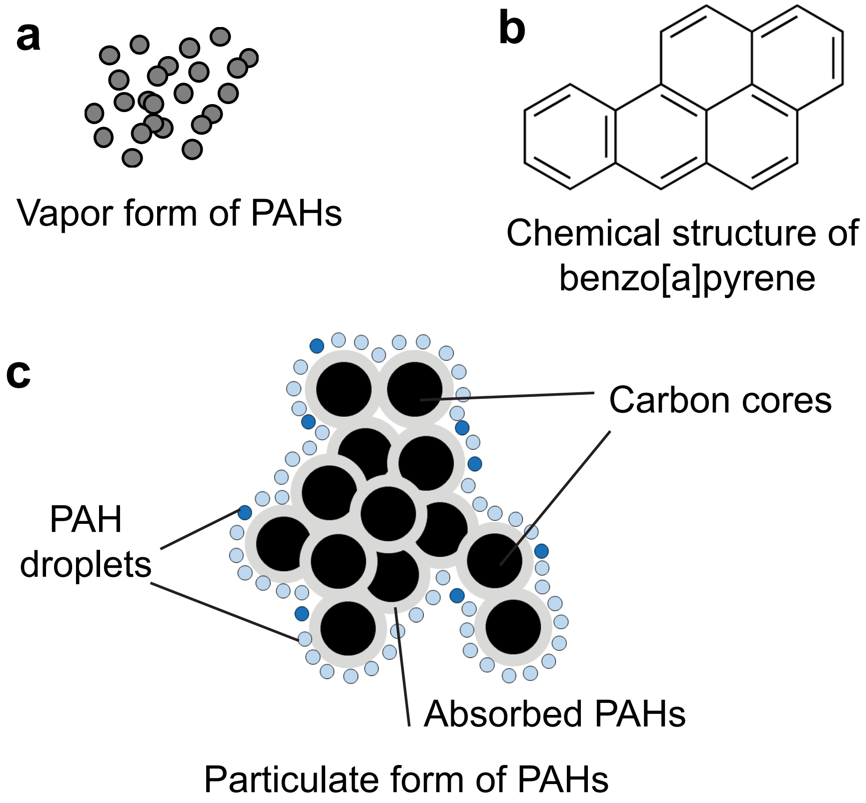

Supplemental Figure 2. Schematic demonstration of the vapor form (a) of polycyclic aromatic hydrocarbons (PAHs) with the structure of benzo[a]pyrene (b) and the particulate form (diesel exhaust particle) of PAHs (c).

a. Primers used in real-time RT-PCR.

Primer	Amplicon size (bps)	Sequence: 5'→3'
IFIT1-F	131	GGA AGG ATG GGC CTT GCT GA
IFIT1-R		GGC GAT AGG CAG AGA TCG CA
IL22RA2-F	174	ACC CAA GTC AAT GGC TCT TTG TT
IL22RA2-R		CGC TCT GTG AGC CCC TTC ATA
CXCL10-F	166	CCT GCA AGC CAA TTT TGT CCA C
CXCL10-R		TCG ATT TTG CTC CCC TCT GGT
PPARG-F	174	CCT CAT GAA GAG CCT TCC AAC T
PPARG-R		GTG GAT CCG ACA GTT AAG ATC ACA
APOE-F	173	ACT GGC ACT GGG TCG CTT TT
APOE-R		CCG GGG TCA GTT GTT CCT CC
GAPDH-F	199	GAA GGT GAA GGT CGG AGT CA
GAPDH-R		GAC AAG CTT CCC GTT CTC AG
B2M-F	70	AAG TGG GAT CGA GAC ATG TAA GCA
B2M-R		GGA ATT CAT CCA ATC CAA ATG CGG

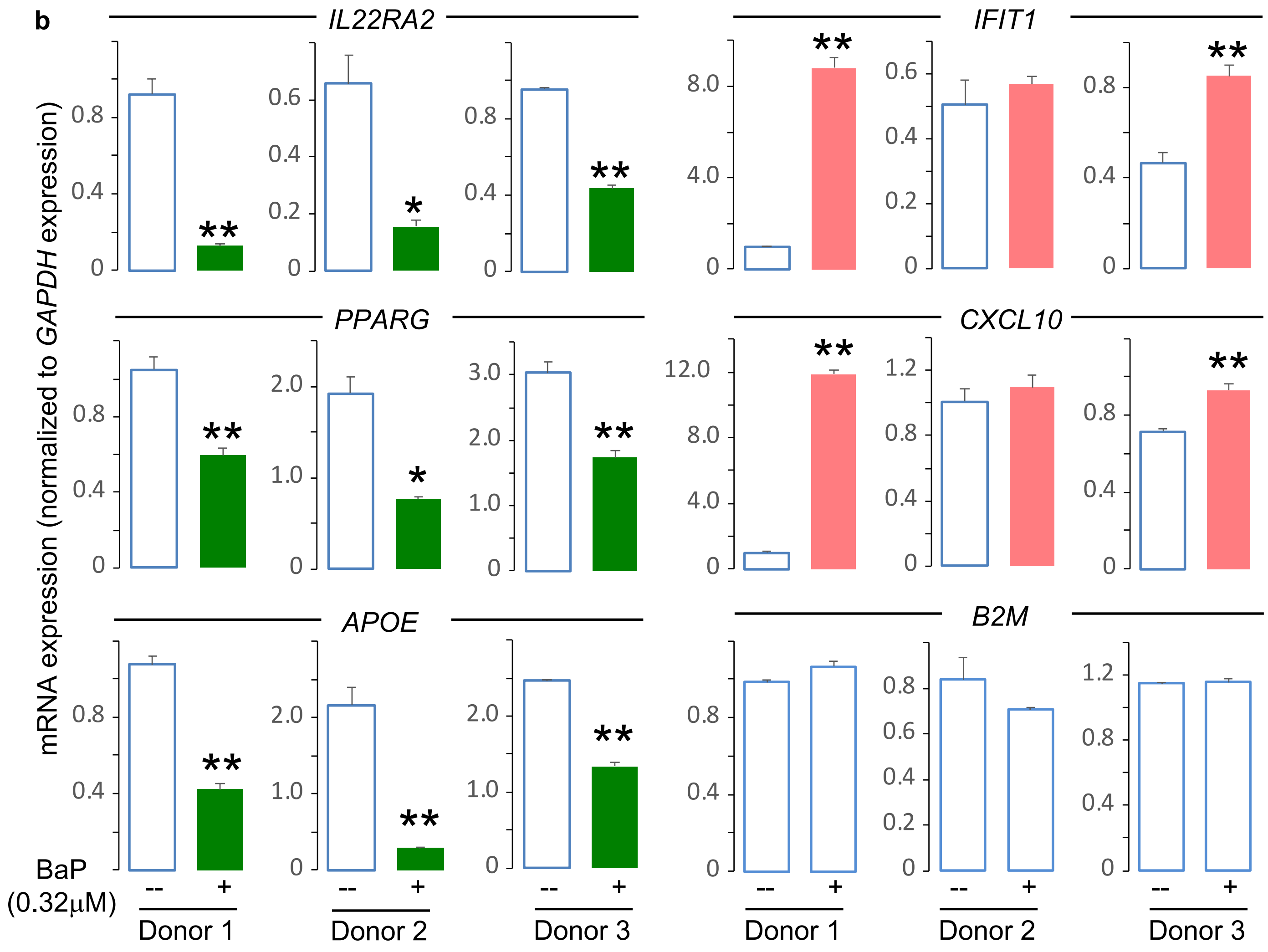

Supplemental Figure 3. Altered gene expression shown by RT-qPCR in BaP-exposed human dendritic cells. (a) primers for targeted gene amplification; (b) the expression level of targeted genes was normalized to that of the *GAPDH* gene. Gene expression in BaP-exposed and non-exposed DCs were compared using three individual donors. The standard errors were calculated from triplicate reactions. The p-values were calculated using student's t-test and shown as ** (P<0.01) or * (P<0.05).

Genes	Description
NCF1	subunit of neutrophil NADPH oxidase
CXCR2	a receptor for interleukin 8
MDM2	nuclear-localized E3 ubiquitin ligase,
ARAP3	phosphoinositide binding protein
OLR1	low density lipoprotein receptor
SMAD3	transcriptional modulator activated by TGF-b
ITGB5	integrin, beta 5
MARCO	macrophage receptor with collagenous structure
CTSB	cathepsin B,
ACP2	lysosomal acid phosphatase
DYNC1I1	dynein cytoplasmic 1 intermediate chain 1
PSD3	pleckstrin and Sec7 domain containing 3
ASAH1	acid ceramidase
MSR1	macrophage scavenger receptors
DNASE2	deoxyribonuclease II, lysosomal
THBS3	Thrombospondin family protein
LAMP1	Lysosomal-associated membrane protein 1
DNAJC6	DnaJ (Hsp40) homolog
SMPD1	phingomyelin phosphodiesterase 1
CTSD	cathepsin D
CXCR4	chemokine receptor
NPC1	Niemann-Pick disease, type C1
GM2A	GM2 ganglioside activator
CTSL	cathepsin L,

Supplemental Figure 4. The list of genes from the clusters (purple) involving encodytic functions in Fig. 3b.

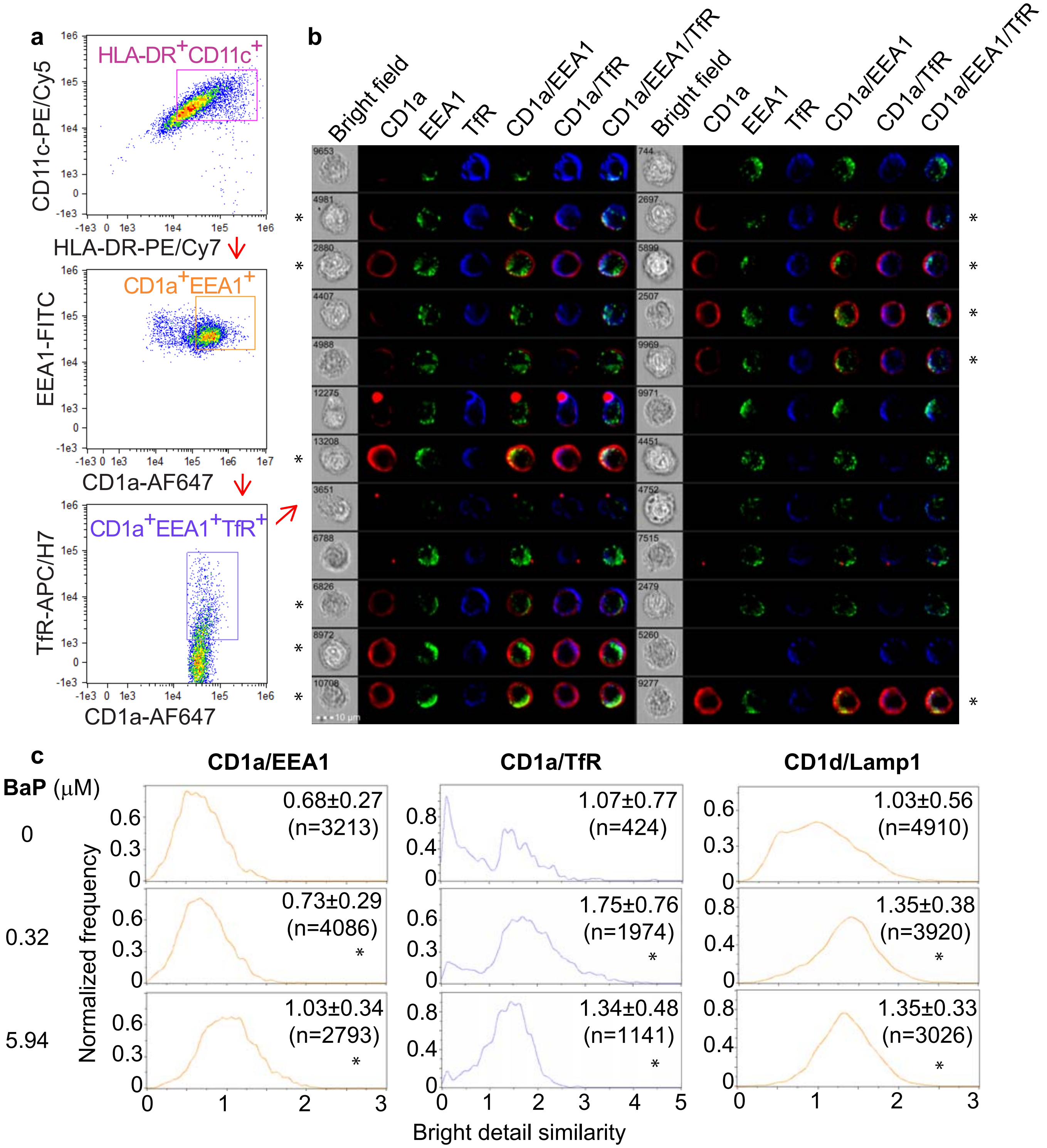

Supplemental Figure 5. Analysis of cellular images using IDEAS software. (a) Monocyte-derived DCs from healthy donors were firstly gated on HLA-DR$^+$CD11c$^+$ cells and further gated on CD1a$^+$EEA1$^+$ cells and CD1a$^+$EEA1$^+$TfR$^+$ cells. (b) Cellular images were extracted from the HLA-DR$^+$CD11c$^+$CD1a$^+$EEA1$^+$TfR$^+$ subset based on two technical criteria: strong co-stain of CD1a, EEA1, and TfR proteins, and subcellular localization of staining. The examples of co-stained cells (*) extracted for further co-localization analysis using ImageJ-Fiji software are shown. Unannotated cells showing invisible or excessive staining, or lack of visual subcellular distribution from some channels at this threshold setting were not extracted. These critieria were applied to all gated cell subsets and all BaP-exposed conditions for co-localization analysis using ImageJ-Fiji software. (c) Bright detail similarity was also calculated using IDEAS software according to the manufacturer's instructions. The mean of bright detail similarity was shown for different BaP-exposed conditions and different combinations of co-localized molecules. Mean ± standard deviation of bright detail similarity and the gated cellular events (n) were shown. Statistical significance ($p<0.0001$) was obtained for BaP-exposed conditions (*) with student's t-tests in comparison to non-exposed conditions. Data are from one experiment using cells from an independent donor. Two experiments were performed with similar results

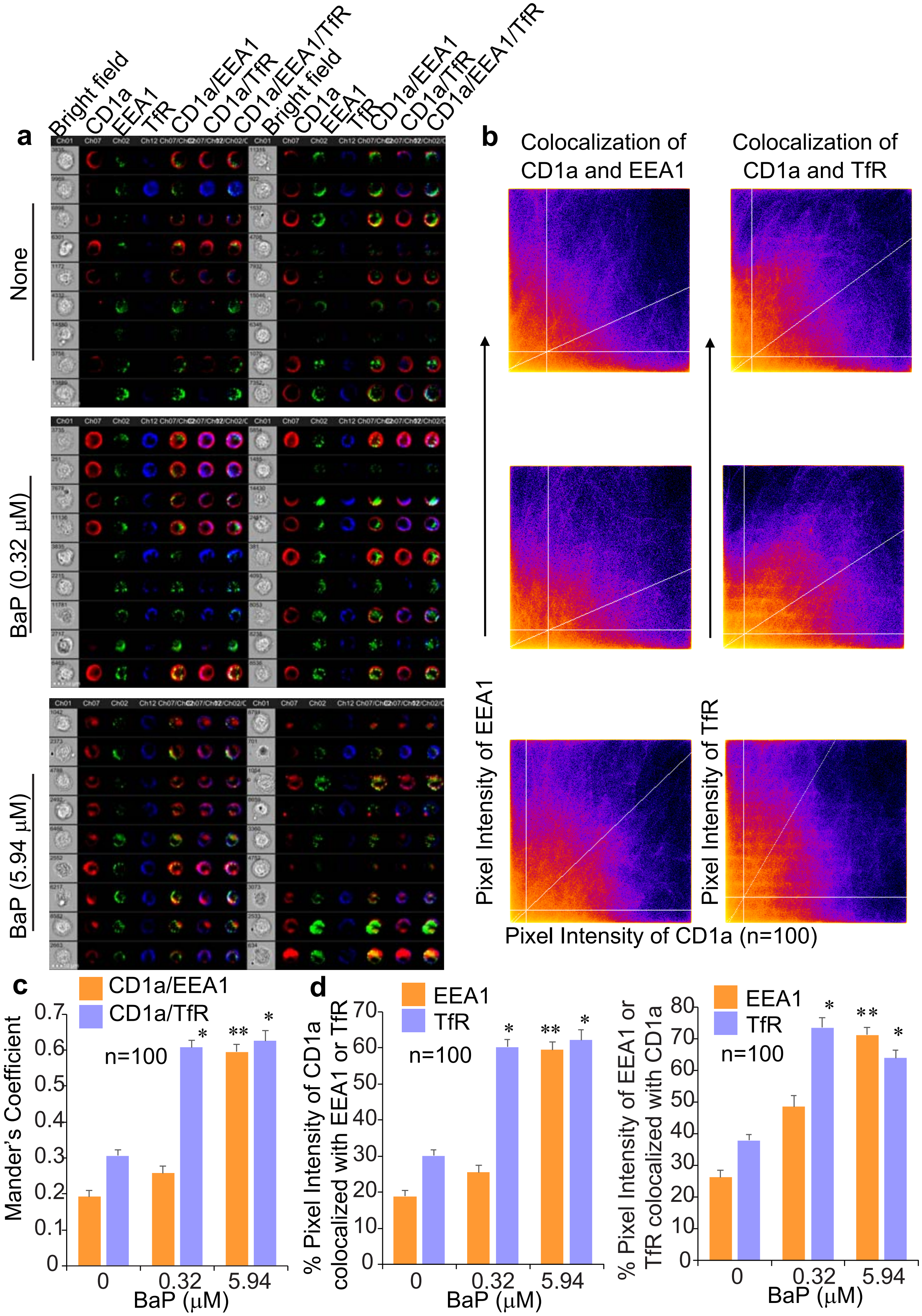

Supplemental Figure 6. CD1a protein retained in early and recycling endosomes using a duplicated analysis with 100 cellular images. Gated $HLA\text{-}DR^{+}CD11c^{+}CD1a^{+}EEA1^{+}TfR^{+}$ population from each BaP-exposed condition are shown with cell identities (numbers) and size bars (a). A hundred of cells with strong co-stain and visible subcellular localization of CD1a, EEA1, and TfR proteins were extracted as exemplified in Supplemental Fig. 5b for analysis using ImageJ-Fiji. The co-localization of pixel intensity between different channels is visualized with scatterplots (b), in which the horizontal and vertical lines represent Costes's thresholds and the diagonal lines represent the ratio of overall pixel intensity between two channels. Moreover, co-localized areas were quantified with the Mander's coefficient between CD1a and EEA1 proteins, and between CD1a and TfR proteins (c). Finally, the percentage of co-localized pixel intensity is also shown (d). Statistical significance ($p<0.001$) was obtained with student's t-tests in comparison to the non-exposed group (*) or together with lower exposure group (**) with indicated standard errors.

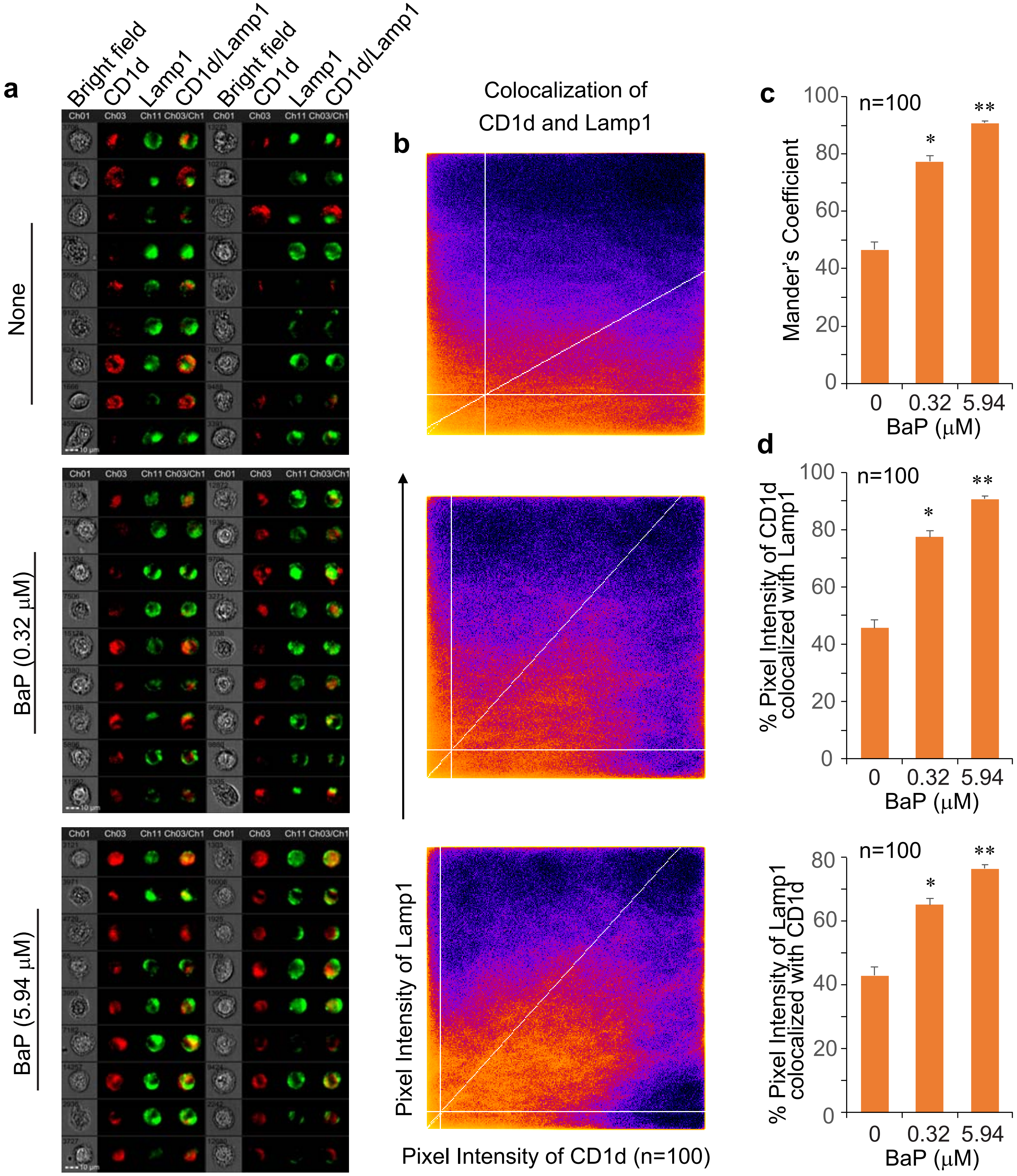

Supplemental Figure 7: CD1d protein retained in late endocytic compartments with a duplicated analysis using 100 cellular images. Cellular images from the gated HLA-DR$^+$CD11c$^+$CD1d$^+$Lamp1$^+$ human DCs are shown together with cell identities and size bars (a). A hundred of cell images with strong co-stain and clear subcellular localization of CD1d and Lamp1 proteins were extracted as exemplified in Supplemental Fig. 5 for analysis using ImageJ-Fiji. The co-localization analyses were shown with scatterplots (b) and quantified with an Mander's coefficient for co-localized areas (c). The percentage of co-localized pixel intensity is also shown (d). Statistical analyses were similarly performed as in Fig. 6.

Supplemental Table 1: Gene expression altered by BaP exposure (0.32mM) in human DCs

Gene_name	Fold_change	Log2(Fold_change)	FDR_adjusted_p
EDA2R	1720.58	10.75	2.31E-20
PADI4	342.85	8.42	4.64E-24
PTCHD4	111.71	6.80	1.63E-03
TSPAN11	66.81	6.06	1.25E-02
CXCL10	44.51	5.48	2.50E-02
CCL1	33.06	5.05	2.52E-02
PLXNA2	25.18	4.65	7.62E-08
TLR10	22.39	4.48	4.11E-02
KRT17	19.62	4.29	6.31E-38
SPATA18	19.03	4.25	1.01E-06
ACTA2	18.02	4.17	6.63E-43
VWCE	17.19	4.10	2.69E-13
GPR162	15.81	3.98	3.75E-02
CD101	15.03	3.91	1.23E-15
GDF15	14.19	3.83	2.37E-08
LRP5	13.90	3.80	4.03E-04
FCRL1	13.87	3.79	1.20E-02
RP11-506M13.3	11.63	3.54	1.15E-02
C7orf57	10.48	3.39	6.80E-08
CXCR2	10.01	3.32	3.89E-03
AIRE	9.74	3.28	2.55E-02
PLK2	8.36	3.06	1.35E-03
TNFSF10	7.20	2.85	1.22E-06
TNFRSF10C	6.95	2.80	5.53E-05
MDM2	6.58	2.72	9.69E-50
PLAT	6.35	2.67	5.23E-05
VSTM2L	6.30	2.66	5.74E-03
FDXR	6.21	2.64	3.22E-04
MLXIPL	6.17	2.63	2.27E-02
ARHGAP6	5.94	2.57	3.52E-05
FAS	5.88	2.56	1.93E-35
PLSCR1	5.61	2.49	4.69E-08
PVT1	5.54	2.47	2.92E-03
VCAN	5.04	2.33	1.24E-05
EGR1	4.85	2.28	1.13E-04
CTD-2547L24.3	4.69	2.23	1.59E-06
CLCN4	4.62	2.21	7.78E-07
MS4A1	4.43	2.15	2.16E-03
DDB2	4.34	2.12	1.05E-28
FAM157C	4.31	2.11	3.82E-02
FAM212B,FAM212B	4.20	2.07	3.54E-14
IFIT1	4.15	2.05	1.34E-03
TRIM22	4.02	2.01	5.43E-29
ZNF530	3.97	1.99	3.75E-02
CYFIP2	3.91	1.97	4.66E-11

RSAD2	3.88	1.95	3.32E-05
ALOX5	3.85	1.94	1.64E-04
SEC14L5	3.81	1.93	2.97E-02
SECTM1	3.75	1.91	1.60E-02
ARAP3	3.66	1.87	4.49E-02
ATP8B4	3.66	1.87	6.40E-08
CMPK2	3.49	1.80	8.10E-04
TAF9B	3.47	1.80	6.60E-05
PPP2R2B	3.47	1.79	3.38E-03
NFATC2	3.46	1.79	7.19E-09
NCF1	3.38	1.76	2.51E-04
CKLF	3.38	1.76	2.82E-03
CDR2L	3.32	1.73	2.71E-09
PCNA	3.31	1.73	7.20E-20
IFIT2	3.29	1.72	8.51E-04
ENC1	3.28	1.71	1.24E-07
IFIT3	3.26	1.70	8.98E-03
FAM189A2	3.23	1.69	3.94E-02
LILRA1	3.16	1.66	9.57E-06
TMEM154	3.15	1.66	3.68E-02
SMAD3	3.15	1.65	4.65E-02
HIC1	3.12	1.64	1.30E-02
RPS27L	3.09	1.63	8.15E-08
ARHGEF3	3.05	1.61	6.39E-26
POLH	3.05	1.61	1.20E-06
EREG	3.03	1.60	1.63E-03
SESN1	3.00	1.59	5.26E-07
SEMA6B	2.99	1.58	2.59E-02
CACNA2D4	2.84	1.51	6.87E-05
LPAR6	2.83	1.50	7.20E-09
AEN	2.82	1.50	7.55E-07
MX2	2.74	1.46	4.13E-02
TPM2	2.71	1.44	4.08E-02
HERC5	2.71	1.44	2.39E-02
RRM2B	2.69	1.43	1.14E-13
PLEKHA6	2.68	1.42	2.37E-02
GCNT1	2.68	1.42	1.69E-04
TIFAB	2.67	1.42	1.84E-09
ZMAT3	2.64	1.40	3.34E-18
CHN2,CHN2	2.64	1.40	2.28E-06
BBC3	2.63	1.39	1.32E-03
ATP5EP2	2.58	1.37	2.26E-02
TMEM30A	2.57	1.36	3.18E-14
ADORA3	2.54	1.34	5.64E-05
MX1	2.53	1.34	2.41E-02
OAS2	2.51	1.33	2.07E-02
ADA	2.51	1.33	4.65E-02

ISYNA1	2.50	1.32	5.48E-10
TVP23A	2.49	1.32	3.15E-02
OLR1	2.48	1.31	4.76E-03
KDELC2	2.46	1.30	2.34E-03
REV3L	2.43	1.28	1.71E-06
DGKA	2.41	1.27	5.35E-06
RAB15	2.40	1.26	3.45E-03
TRIAP1	2.39	1.25	1.08E-07
APOBEC3C	2.35	1.23	6.01E-13
RCBTB2	2.33	1.22	1.41E-12
PHLDA3	2.31	1.21	1.22E-03
PHPT1	2.31	1.21	3.15E-02
WWC3	2.28	1.19	3.48E-07
ICAM3	2.28	1.19	3.45E-03
SAMD9	2.27	1.18	1.72E-04
NLRP3	2.26	1.17	2.02E-03
FGL2	2.21	1.14	4.43E-10
STXBP5	2.20	1.14	7.58E-11
CTD-2228K2.7	2.19	1.13	2.16E-04
TIPARP	2.19	1.13	6.41E-06
EPHB2	2.18	1.13	1.89E-02
F3	2.18	1.13	1.07E-03
FA2H	2.18	1.13	5.16E-03
MMP12	2.17	1.12	7.82E-07
LINC00926	2.17	1.12	5.51E-04
TBL1X	2.16	1.11	1.34E-03
RGS12	2.13	1.09	6.09E-05
PLEKHG5	2.13	1.09	3.35E-08
SESN2	2.13	1.09	1.91E-04
GIN1	2.12	1.09	6.48E-03
XAF1	2.09	1.07	2.37E-02
DDX60L	2.09	1.06	6.03E-03
TMEM45B	2.08	1.06	1.27E-05
PDP1	2.08	1.06	7.86E-03
STK38L	2.07	1.05	1.11E-06
EPB41L3	2.07	1.05	9.39E-07
PTP4A1	2.07	1.05	4.26E-08
SLC30A4	2.06	1.05	6.31E-04
CCND2	2.06	1.04	1.54E-05
DEF6	2.05	1.04	2.42E-03
EPSTI1	2.05	1.03	4.29E-02
NET1	2.05	1.03	1.01E-06
CASS4	2.04	1.03	4.53E-05
RASSF5	2.04	1.03	9.02E-10
ZNF107	2.03	1.02	3.50E-02
PIDD1	2.03	1.02	1.85E-02
CDH2	2.03	1.02	1.05E-02

IFI44	2.03	1.02	3.98E-02
TNFRSF10B	2.02	1.02	6.17E-08
NPC1	0.50	-1.00	1.46E-05
ADPGK-AS1	0.50	-1.00	2.76E-02
PPP2R3A	0.50	-1.00	2.74E-05
RABEPK	0.50	-1.00	2.69E-07
LAMP1	0.50	-1.01	2.28E-06
ABHD4	0.50	-1.01	3.33E-05
SLC26A11	0.50	-1.01	7.94E-04
RP11-701P16.5	0.49	-1.02	2.18E-02
ECHDC3	0.49	-1.03	4.40E-02
RRAGD	0.49	-1.03	6.39E-05
SCD	0.49	-1.03	1.01E-08
SLC1A3	0.49	-1.03	2.74E-05
TNFAIP6	0.49	-1.03	1.98E-02
METTL21B	0.49	-1.03	1.03E-05
TXNDC16	0.49	-1.03	9.22E-04
MACC1	0.49	-1.04	5.61E-03
MGLL	0.49	-1.04	5.98E-05
CAMK2D	0.48	-1.05	1.17E-07
AKR1B1	0.48	-1.05	7.31E-08
TSPAN7	0.48	-1.05	1.55E-02
KIAA1161	0.48	-1.06	4.38E-06
ASAH1	0.48	-1.06	8.49E-05
MS4A6A	0.48	-1.06	3.06E-07
NPL	0.48	-1.06	3.01E-08
UBBP4	0.48	-1.07	1.10E-02
AK4	0.48	-1.07	6.62E-03
RAB33A	0.48	-1.07	7.39E-04
CPM	0.48	-1.07	1.09E-10
NPHP4	0.48	-1.07	3.94E-02
CYP27A1	0.48	-1.07	1.05E-04
MARCKS	0.47	-1.08	8.56E-04
SLC25A35	0.47	-1.08	2.40E-04
MEX3B	0.47	-1.08	3.21E-03
CCL22	0.47	-1.08	2.05E-07
HS6ST1	0.47	-1.09	1.22E-05
MARCH9*	0.47	-1.09	3.41E-02
TMPRSS13	0.47	-1.09	1.19E-02
AHNAK2	0.47	-1.09	5.31E-05
NHSL1	0.47	-1.10	4.14E-02
CCL13	0.47	-1.10	3.23E-04
FTCDNL1	0.47	-1.10	4.08E-02
SMPD1	0.46	-1.11	1.99E-06
HYAL3	0.46	-1.11	4.54E-03
MARCH3*	0.46	-1.11	3.17E-03
CCND1	0.46	-1.11	1.28E-06

FZD7	0.46	-1.11	1.44E-02
TNS3	0.46	-1.12	2.82E-11
TAGLN	0.46	-1.13	1.60E-02
WFS1	0.46	-1.13	4.43E-06
HLA-J	0.46	-1.13	4.11E-02
CTSB	0.46	-1.13	1.55E-08
CHI3L1	0.46	-1.13	4.13E-05
C5AR1	0.46	-1.13	2.37E-02
SYT1	0.46	-1.13	4.89E-06
CD59	0.46	-1.13	1.84E-07
GM2A	0.46	-1.13	3.10E-07
GJB2	0.45	-1.14	9.69E-04
IL18	0.45	-1.14	8.09E-04
NRP2	0.45	-1.14	3.03E-11
RP11-134L10.1	0.45	-1.14	2.07E-02
STEAP3	0.45	-1.15	1.81E-04
ITGA6	0.45	-1.15	2.82E-05
LGR4	0.45	-1.15	1.72E-06
ST6GAL1	0.45	-1.15	6.46E-12
NOTCH3	0.45	-1.15	8.56E-04
SAMD4A	0.45	-1.15	1.10E-05
ABCG2	0.45	-1.16	1.14E-05
PPP1R16B	0.45	-1.16	6.01E-13
STRIP2	0.45	-1.16	8.70E-03
FAM20A	0.45	-1.16	2.38E-07
CHST7	0.45	-1.16	7.29E-05
CD22	0.45	-1.17	5.21E-03
STMN1	0.44	-1.18	1.51E-02
DTX4	0.44	-1.18	5.26E-03
LRWD1	0.44	-1.19	3.94E-02
MFI2	0.44	-1.20	1.01E-03
RP5-839B4.8	0.43	-1.20	5.42E-03
ARID5B	0.43	-1.21	2.13E-06
PC	0.43	-1.21	1.70E-07
CR1	0.43	-1.21	7.19E-09
PPARG	0.43	-1.22	2.42E-05
FABP4	0.43	-1.22	1.60E-10
MAP1A	0.43	-1.23	4.13E-08
SLC18A2	0.42	-1.24	9.48E-07
TUT1	0.42	-1.26	3.50E-02
MSR1	0.42	-1.27	5.40E-03
SLC7A7	0.42	-1.27	8.56E-10
SMAD6	0.42	-1.27	3.86E-03
HSD11B1	0.42	-1.27	2.38E-08
RP11-1008C21.1	0.41	-1.27	1.15E-02
MMP14	0.41	-1.28	6.49E-05
EPB41L1	0.41	-1.28	2.31E-05

DYSF	0.41	-1.28	1.83E-07
SH3BP5	0.41	-1.28	6.08E-04
DCSTAMP	0.41	-1.28	1.45E-06
DNASE2	0.41	-1.29	3.33E-02
S100A9	0.41	-1.29	4.81E-03
CTSD	0.41	-1.30	2.35E-06
PAPSS2	0.41	-1.30	1.58E-04
RAB32	0.41	-1.30	1.62E-11
CCDC85C	0.40	-1.30	8.10E-03
SYNGR1	0.40	-1.31	3.70E-02
SLC2A8	0.40	-1.31	4.14E-02
DEXI	0.40	-1.31	4.39E-02
CLU	0.40	-1.32	1.74E-03
PMP22	0.40	-1.32	8.07E-06
RAB42	0.40	-1.32	8.50E-04
PFKM	0.40	-1.33	4.96E-03
KIAA1462	0.40	-1.33	8.56E-10
ADGRB1	0.40	-1.33	2.65E-03
CXCR4	0.39	-1.35	5.51E-09
CDS1	0.39	-1.35	2.28E-02
TNIK	0.39	-1.35	4.38E-06
SULT1C2	0.39	-1.36	6.73E-03
ASTN2	0.39	-1.36	2.07E-03
TTN	0.39	-1.36	2.02E-02
MARCO	0.39	-1.36	4.69E-04
CACNA1G	0.39	-1.37	6.08E-03
LHFP	0.39	-1.37	1.24E-07
SLC1A2	0.39	-1.37	1.76E-05
ITGB5	0.39	-1.37	1.32E-03
C9orf139	0.39	-1.37	4.17E-03
FAM110B	0.38	-1.39	5.06E-09
THBS3	0.38	-1.39	9.48E-07
DHCR7	0.38	-1.39	3.27E-06
IGHE	0.38	-1.40	4.99E-02
PDE3B	0.37	-1.43	4.13E-07
SLC12A8	0.37	-1.43	3.55E-02
IL1RN	0.37	-1.44	2.14E-12
ACP2	0.37	-1.45	2.96E-13
CTA-363E6.1	0.36	-1.46	8.02E-05
VCAM1	0.36	-1.47	2.60E-02
PSD3	0.36	-1.48	1.10E-05
GAL	0.36	-1.49	1.30E-02
SYT6	0.36	-1.49	1.77E-02
CLDN23	0.36	-1.49	1.25E-08
BCAT1	0.35	-1.50	1.56E-05
RAPGEF3	0.35	-1.50	4.28E-04
PVRL1	0.35	-1.51	1.82E-09

PCSK5	0.35	-1.51	1.84E-09
MT1E	0.35	-1.52	4.75E-03
CYP19A1	0.35	-1.53	4.86E-02
ZBED6CL	0.34	-1.54	2.41E-04
PRRT3	0.34	-1.54	5.93E-04
NFIA	0.34	-1.56	1.94E-02
SIGLEC15	0.34	-1.56	2.96E-04
RTN4R	0.34	-1.56	5.04E-05
PDIA5	0.34	-1.56	3.67E-03
IFITM10	0.33	-1.60	7.73E-03
UCHL1	0.33	-1.61	1.27E-08
MANEAL	0.33	-1.62	1.50E-04
RNASE1	0.32	-1.64	9.26E-04
LINC00607	0.32	-1.64	1.46E-03
GRB10	0.32	-1.65	1.25E-02
MT1F	0.32	-1.66	9.22E-04
CTSL	0.32	-1.67	9.97E-09
AFAP1L1	0.31	-1.67	3.62E-04
WBP5	0.31	-1.69	4.55E-02
ITGAD	0.31	-1.69	2.00E-02
AC004988.1	0.31	-1.70	1.40E-03
ST8SIA6	0.31	-1.71	5.14E-04
TTC28	0.31	-1.71	6.10E-03
CYP27B1	0.30	-1.73	5.45E-10
DNAJC12	0.30	-1.74	3.88E-03
WWOX	0.30	-1.74	1.85E-02
EPDR1	0.30	-1.75	1.05E-09
HHLA2	0.29	-1.77	1.46E-05
RBFOX2	0.29	-1.80	4.12E-02
RPS6KA2	0.29	-1.81	1.76E-05
C1S	0.28	-1.81	1.33E-02
ADAMTS15	0.28	-1.82	1.46E-03
GGH	0.28	-1.83	1.71E-02
ZRANB3	0.28	-1.84	1.74E-02
DTNB	0.28	-1.85	4.77E-02
ROR1-AS1	0.28	-1.86	1.61E-11
CD163	0.27	-1.88	7.35E-05
DPYSL3	0.27	-1.89	1.50E-02
LINC00520	0.26	-1.92	5.77E-08
NR1H3	0.26	-1.92	7.91E-19
COL5A3	0.26	-1.97	4.44E-04
SPARC	0.25	-1.99	7.49E-05
PAM	0.25	-1.99	5.59E-09
DNAJC6	0.25	-2.02	5.54E-04
FAM101B	0.25	-2.03	1.96E-03
RARRES1	0.25	-2.03	1.91E-03
IL22RA2	0.24	-2.05	6.17E-08

CABLES1	0.24	-2.06	3.78E-03
AKR1C1	0.24	-2.06	2.07E-08
EGLN3	0.24	-2.07	7.77E-08
NCKAP5	0.24	-2.08	9.97E-06
SHROOM3	0.24	-2.09	7.10E-03
APOE	0.23	-2.09	8.41E-04
TNIP3	0.23	-2.09	2.60E-02
DNASE1L3	0.22	-2.16	2.96E-11
FAR2	0.21	-2.23	5.75E-11
CCL26	0.21	-2.27	9.13E-03
ELOVL7	0.20	-2.32	8.07E-03
ABCC2	0.19	-2.36	6.27E-07
APBB2	0.19	-2.38	4.45E-06
CCL23	0.19	-2.40	2.26E-09
HS3ST2	0.19	-2.41	6.51E-07
ALDH1A1	0.19	-2.42	1.91E-06
AC007743.1	0.19	-2.43	4.87E-02
SLC28A3	0.18	-2.46	1.12E-06
ME3	0.17	-2.54	1.20E-06
CES1	0.17	-2.57	7.90E-05
DNAJC5B	0.17	-2.60	6.34E-05
AKR1C3	0.16	-2.69	2.37E-04
CCL18	0.16	-2.69	2.10E-04
CCL15	0.15	-2.74	1.56E-05
GCNT3	0.15	-2.75	1.34E-02
NBEA	0.15	-2.76	5.02E-03
ZNF575	0.14	-2.80	2.96E-02
GS1-600G8.5	0.13	-2.93	7.31E-29
CHDH	0.13	-2.95	3.08E-03
DAAM2	0.11	-3.13	1.26E-02
COBL	0.11	-3.15	1.17E-07
CYYR1	0.11	-3.23	4.55E-03
TMEM200A	0.11	-3.24	3.08E-02
MT1G	0.11	-3.24	2.11E-05
DYNC1I1	0.10	-3.26	1.31E-02
C8orf46	0.09	-3.50	3.02E-02
LINC00639	0.08	-3.56	4.04E-04
HSD3BP5	0.08	-3.60	1.63E-03
PBX4	0.08	-3.65	7.15E-03
WASF3	0.08	-3.72	4.70E-02
RP4-644L1.2	0.07	-3.78	3.35E-02
RN7SKP26	0.07	-3.81	3.64E-02
FAM19A4	0.07	-3.84	6.49E-05
SDK1	0.05	-4.27	3.18E-02
PTPRM	0.05	-4.42	1.02E-04
ANK2	0.00	-8.58	1.68E-08
AC069363.1	0.00	-8.70	4.46E-05
